# Investigating patient navigator impact on older adults’ transitions from acute care: A randomized controlled trial with embedded qualitative component

**DOI:** 10.1371/journal.pone.0341251

**Published:** 2026-02-04

**Authors:** Natasha Hanson, Tracy Freeze, Leanne Skerry, Kathleen O’Keefe, Chi Nguyen, Ravneet Somal, Karla Faig, Pamela Jarrett

**Affiliations:** 1 Research Services, Horizon Health Network, Saint John, New Brunswick, Canada; 2 University of New Brunswick, Saint John, New Brunswick, Canada; 3 Dalhousie University, Dalhousie Medicine New Brunswick, Saint John, New Brunswick, Canada; Instituto Sirio-Libanes de Ensino e Pesquisa, BRAZIL

## Abstract

**Background:**

Fall-related injuries such as fractures are on the rise among older adults. Resulting transitions in care from hospital can be complex and place significant pressure on healthcare teams. This study sought to determine the impact of patient navigators in the orthogeriatric context.

**Methods:**

A concurrent embedded mixed methods design was used, in which the quantitative, open label, randomized controlled trial, analysed using structural equation modelling, had an embedded reflexive thematic analysis qualitative component. Older adults (≥65 years) admitted to an orthopedic unit with any fracture were randomized into the standard of care or patient navigator group and followed until three months post-discharge. Main outcomes were, unplanned healthcare utilization post-discharge, patient satisfaction, and patient/caregiver experiences. Semi-structured interviews were conducted with patients (*n* = 60) and caregivers (*n* = 15) in both groups.

**Results:**

The patient navigator group (*n* = 34) had fewer unplanned healthcare utilizations post-discharge at lower levels of frailty and more unplanned healthcare utilizations post-discharge as frailty increased compared to the standard of care group (*n* = 36). Patient satisfaction was not significantly different between groups. Comparisons between the standard of care and patient navigator patient and caregiver groups found the patient navigator groups had unique themes, detailing the positive impact of the patient navigator, particularly in relation to the provision of information and support.

**Conclusions:**

Patient navigators play an important role in orthogeriatric rehabilitation by facilitating more appropriate use of healthcare resources. Patients and caregivers found patient navigators supportive and helpful with care. Patient navigators were shown particularly helpful for participants with higher care needs and would also be beneficial for patients with fewer family supports. **Trial Registration:** NCT06107699

## Introduction

With Canada’s aging population [1], it is crucial that health and social services are responsive to older adults’ needs. Older adults’ transitions in care from hospital are a critical junction in their healthcare journey, whereby great vulnerability to disparities in health outcomes exist [2–5]. The transitional process is complicated by the multitude of activities surrounding hospital discharge, such as the activation of varying hospital and community resources to facilitate the transition [6,7]. Supporting older adults’ transitions out of hospital can place significant pressure on healthcare teams who are responsible for identifying needs and coordinating care upon discharge. This is especially true for hospital-based healthcare providers (HCPs) working with frail older adults who often have underlying complex medical, cognitive, functional, and social needs. A recent study indicated many patients and families found transitioning from hospital to home a daunting process; thus, guidance and open communication are needed to facilitate these transitions [8,9]. Fortunately, research suggests that patient education and follow-up can be effective in reducing patient readmissions [10,11] as a component of orthogeriatric care [12].

Patient navigators (PNs) are a patient-centred approach to providing education and follow up, as well as coordinating care and discharge with health and social care providers [13]. The main goal of PNs is to guide patients through health and social care systems to improve health outcomes and timely access; thereby, reducing socio-economic, informational, and logistical barriers [13–15]. PNs could play a pivotal role in orthogeriatric rehabilitation by helping patients identify and access appropriate services/resources; thus, facilitating seamless transitions to community.

Patient populations who may benefit most from PNs are those with complex needs, including frailty, and/or populations negatively impacted by social determinants of health [16,17]. PN programs are typically employed in the context of cancer or other illnesses whereupon healthcare delivery may be fragmented between different providers and healthcare settings [13]. As such, PNs may be particularly important for frail older adults who often have multiple HCPs in different settings, whereby discontinuity in care and poor communication may exist [18,19]. Older adults who have recently been hospitalized, such as for a fracture, are an example of a complex population that might benefit from PNs. The literature suggests that interventions designed to improve care coordination and communication in the inpatient setting and after discharge, may be effective in reducing readmissions [20,21] to enable successful aging in place. Additionally, PNs for patients at risk of readmission have decreased readmission rates for participants aged 60 or older [22,23], and increased satisfaction with care [24]. PNs can support patients and families in accessing necessary resources and services during transitions in care; thereby, facilitating timely transitions and fostering self-management [25,26].

Fall-related injuries, such as fractures, often lead to adverse health outcomes for older adults, including increased frailty, admission to nursing homes or assisted living facilities, and even death [27–29]. After a fall, older adults treated in hospital should be supported so that as many as possible can return to the community.

The overall goal of this project was to investigate effects of the implementation of PNs, working alongside the usual healthcare team on an Orthopedic Unit (OU) in one hospital in New Brunswick, targeting adults aged 65 and older admitted with a fracture. Specifically, a mixed methods analysis was used to describe the difference between older adults who received PNs compared to those who received usual standard of care (SOC). The main outcomes included unplanned healthcare utilization post discharge (UHU), patient satisfaction (PS), and patient/caregiver experience.

## Materials and methods

### Study design

This study used a concurrent embedded mixed methods design, in which the quantitative open-label, randomized controlled trial had an embedded qualitative component [30]. No patients or public were involved in the design, conduct, or reporting of the study. The parallel randomized controlled trial was conducted in one inpatient OU at one New Brunswick hospital. The duration of the project was June 2022 to December 2024. The recruitment period was 01/09/2022–03/10/2023. The study was registered with ClinicalTrials.gov (NCT06107699). Ethics approval was obtained through the Horizon Health’s Human Research Protection Program, inclusive of the Horizon Health Network Research Ethics Board.

### Participants

Potential patient participants were approached by HCPs in their circle of care, to see if they were interested in participating in the study, if they met the following eligibility criteria: 1) 65 years or older; 2) English-speaking; and, 3) admitted to the research site OU with any fracture. If a patient was interested, this information was relayed to the research team, who would contact the patient/substitute decision-maker (SDM)with project information and provide them with the consent form. Written informed consent was obtained from either the patient or their SDM with patients who had SDMs assenting to be part of the research study. Patients who agreed to participate were asked to identify a family caregiver if they would like one to be approached to participate in the research study. Family caregivers were asked by a research team member if they would also like to participate in the study and provided with the appropriate consent form. Family caregiver participants also provided written informed consent. Informed consent was obtained by a research team member before any data collection began.

### Intervention and comparator

The intervention consisted of the provision of a PN from the time of consent to 3 months post-discharge from the OU. Patient Navigators all had prior healthcare provider experience and prior to the start of recruitment obtained formal patient navigation training and certificate from a Canadian university. The role of the PNs was to assess patients, co-develop personalized transition goals with patients/SDMs, and facilitate these transition goals, as well as adapt them to new patient needs over the course of the intervention. PNs conducted a Comprehensive Geriatric Assessment (CGA) [31,32] which informed personalized goals for each patient (S2 File, Appendix A). The CGA entailed gathering information regarding medical, functional, psychological, and social assessments from the patient and/or family members and HCPs. The CGA allowed for the development of a care plan needed for a successful discharge, and was patient specific, attainable, and focused on the following areas: Patient and family engagement, education and collaboration; Healthcare needs identified that needed ongoing care and follow up after discharge; Pre-existing healthcare needs; Fracture related healthcare needs; fall risk assessment; Prevention strategies that needed to be put in place prior to discharge; Functional (Activities of Daily Living and Instrumental Activities of Daily Living) needs identified and how they would be met at the time of discharge; Social care needs that needed to be in place prior to discharge; Appropriate connection to community resources as required; Appropriate communication to primary care provider and institutions as needed at the time of discharge; and, Plans for needed follow up with Orthopedics and primary care providers. Resources compiled by the PNs is available as a PN Toolkit online (https://horizonnb.ca/wp-content/uploads/2025/01/Horizon_PN_Toolbox.pdf). Patients had their assigned PNs contact information, to reach out to at any time through the study period. PNs also ensured regular contact with patients to assist with their patient navigation needs, by setting cooperatively with each patient a regular interval and means of contact through the study period.

The comparator was usual standard of care, wherein patients were provided with discharge information from OU healthcare providers and assisted by discharge planners within the hospital when appropriate. The PN intervention group also received this usual care.

### Outcome measures

All baseline patient data were collected in-person. Patient descriptives (age, gender, ethnicity, education level, and marital status) and level of frailty, using the Pictorial Fit-Frail Scale-Acute (PFFS) [33,34] as well as usual frailty (recalled frailty prior to hospitalization) were collected upon consent (see https://www.dal.ca/sites/gmr/our-tools/pictorial-fit-frail-scale.html for scale examples). The PFFS form was sent home with patients and was also collected in-person or via telephone at discharge (defined as within 72 hours of hospital discharge), and three months post-discharge. The PFFS is a practical, picture-based assessment of frailty in older adults [33]. The PFFS assesses 14 categories ranging from mood to bladder control. Each category is represented by a series of pictures corresponding to levels of impairment. Total scores can range from 0–43 with 43 representing severe frailty and 0 representing no frailty. The PFFS has been shown to have good test-retest and inter-rater reliability between nurses and geriatricians [35,36], content validity [33], and concurrent validity [37]. There are several versions of the PFFS allowing for use by either clinician or layperson and measurements of current fitness-frailty, usual-state fitness-frailty, or both. Because this research required both current and usual-state fitness-frailty measurement, we used the PFFS-Acute version, which took approximately 5 minutes for patients/SDMs to complete.

The co-primary quantitative outcome measures were UHU and PS. Participants were given a form to track UHU after discharge and were contacted via telephone monthly to gather these data with the final collection being 3 months post discharge. UHU consisted of the number of nights they remained in hospital in another hospital bed after OU discharge, the number of emergency department visits, and the number of nights spent in hospital following an unplanned admission. Three months post-discharge from the OU, patient participants were asked one Likert scale survey question on overall patient healthcare satisfaction (PS). One global question was asked on the telephone to patients: “Thinking about all of your healthcare experiences related to your fracture in the last 3 months, to what degree are you satisfied or dissatisfied with the services you have received? Use any number from 0 to 10, with 0 representing the least possible satisfaction with your healthcare experiences and 10 representing the most possible satisfaction with your healthcare experiences.” This question was adapted from the global hospital rating in the CPES-IC.

#### Qualitative data collection.

Three months post-discharge from the OU, research team members who did not participate in the intervention, were not involved in patient care (CN, RS, KO, NH), and were trained in qualitative methods, conducted semi-structured interviews with patients who did not have a SDM and family caregiver participants about their experiences transitioning from acute care. Semi-structured interviews were conducted, and audio recorded either via telephone or in-person, as per participant preference. The decision as to whether to participate in these semi-structured interviews one-on-one or together (i.e., patient and family caregiver) was left to patients and family caregivers to make. Patients and/or family caregivers in both the PN and SOC groups were asked about their experiences in transitioning from the OU. Those participants in the intervention group were asked about their experiences with the patient navigator. All participants were asked about any improvements that could be made regarding transitional care (see S2 File, Appendix D, Participant Semi-Structured Interview Guides). Semi-structured interviews were transcribed verbatim and deidentified.

### Sample size

**Fig 1:**
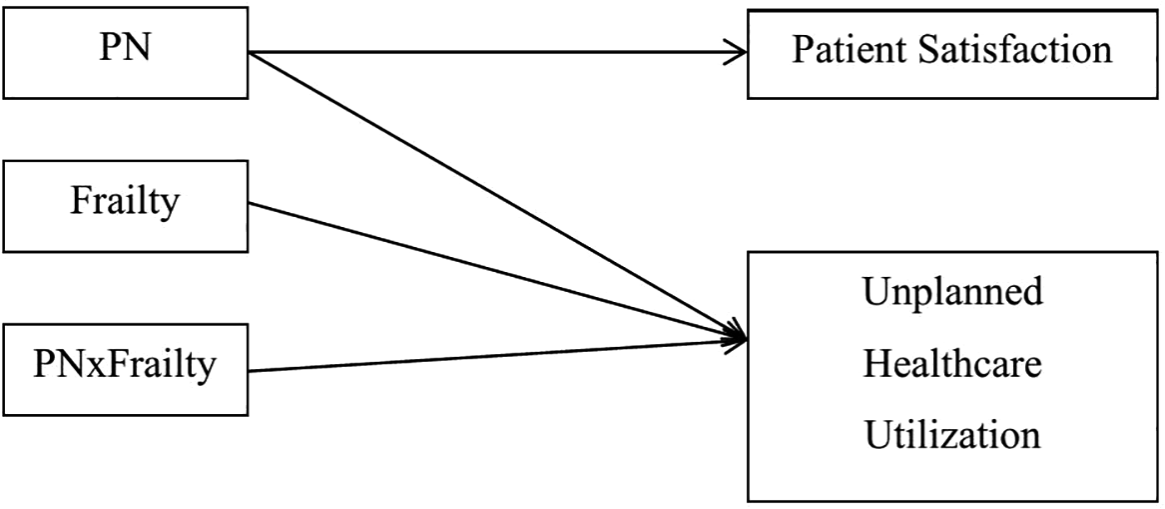
Structural equation model of patient navigator vs. standard of care and frailty predicting unplanned healthcare utilization and patient navigator vs. standard of care predicting patient satisfaction.

A series of Monte Carlo simulations were conducted to estimate the sample size needed to observe the PN effect on UHU and PS during the three months following OU discharge. A model of these relationships are illustrated in Fig 1. Comparable studies reporting quantitative data and effect sizes were lacking; thus, moderately conservative effect sizes were simulated. With the exceptions of the relationship between frailty and UHU (*d* = .5) [37–40], a small to medium effect size (*d* = 0.35) was used for simulations of these primary relationships. Simulations were conducted in MPlus © 8.10, and the sample size was selected based on the following criteria: no warnings or error messages were found in the output, the 95% cover was between.91 and.98, parameter bias was less than or equal to 5%, standard error bias was less than or equal to 5%, and % sig coeff was.8 [38]. A total sample size of 60 patients or 30 patients per group was estimated to be necessary to obtain an approximate power of approximately.8 and alpha of.05 for the study, though a larger sample size was preferable to ensure stability of the model during estimation and to reduce the chance of Type II error. Minimum sample size requirements were met.

For the qualitative component, we aimed to conduct semi-structured interviews with all patients without an SDM and all family caregivers. The sample size was found to have sufficient information power to complete the research study [39].

### Randomization, allocation concealment, and blinding

Patients who met the inclusion criteria were consented and assigned to either the PN group or SOC group using simple randomization via an online generator (RANDOM.ORG - List Randomizer) and a 1:1 ratio. The research team was made aware of participant allocation prior to consent, to ensure PN group baseline data was collected. However, patients were approached by HCPs from within their circle of care who were not aware of the participant randomization, as such enrollment to a particular group could not be influenced. Research team members conducting informed consent were responsible for advising participants as to group allocation. Blinding was not possible, as this was an open-label trial; thus, increasing the likelihood of post-randomization confounding. For instance, patients assigned to the SOC group may report lower satisfaction and less positive experiences simply because they know they did not receive the care of the PN. However, bias due to factors such as non-compliance were minimized by using intention-to-treat analysis. All participants were analyzed in their originally assigned groups regardless of whether they complied with the PN plan or dropped out of the study altogether.

### Data analysis

#### Quantitative.

Study variables and participant demographics were summarized with descriptive statistics. Mplus version 8.1.0 was used to examine a structural equation model where: 1) intervention group (PN or SOC) was expected to predict PS; 2) PFFS at time of consent was expected to predict UHU; and, 3) intervention group was expected to impact the relationship between PFFS and UHU (see Fig 1). This modeling approach allows simultaneous estimation of relationships among predictors and outcomes, which is standard practice in SEM and avoids inflated type I error associated with conducting separate univariate tests [40]. The SEM framework thus provides a cohesive test of the model rather than separate independent outcome tests.

Data were checked for data entry errors. During the 3-month follow-up, three participants withdrew from the SOC group and three participants withdrew from the PN group. The six withdrawn participants were missing 50% of their data and together represented 8.5% of the total sample size. The 8.5% missing in each of the outcome variables exceeds the rule of thumb cut-off (~5%) that would allow the missing data to be considered ignorable. We initially intended to conduct missing data analysis with model-based multiple imputation following Enders’ process for missing data [41]. However, the missing data was too large in proportion case wise (>40%) and represented missingness of both dependent variables. Therefore, multiple imputation could not be used and only observed data were analyzed as recommended by Jakobsen et al. [42], resulting in a sample size of 70. Though the limitations of using only observed data include the loss of statistical power, this should be minimized as our sample size requirements (*n* = 60) were met.

#### Qualitative.

Transcripts were uploaded to NVivo, a qualitative analysis software, and data for each participant group were separately subjected to iterative reflexive thematic analysis [43] and then compared. Team analysis was conducted by qualitatively trained research team members (KO, CN, RS, LS, NH), and themes were agreed upon through consensus. Team members met regularly during iterative thematic analysis, while interviews were still being conducted, to reflexively discuss their coding.

#### Data integration.

Integration occurred at the interpretation phase, in which quantitative and qualitative results were analysed separately and then compared by the research team to explore convergence, divergence, and complementary insights [30]. This integration is reported within the Discussion section.

## Results

### Participant characteristics

**Fig 2:**
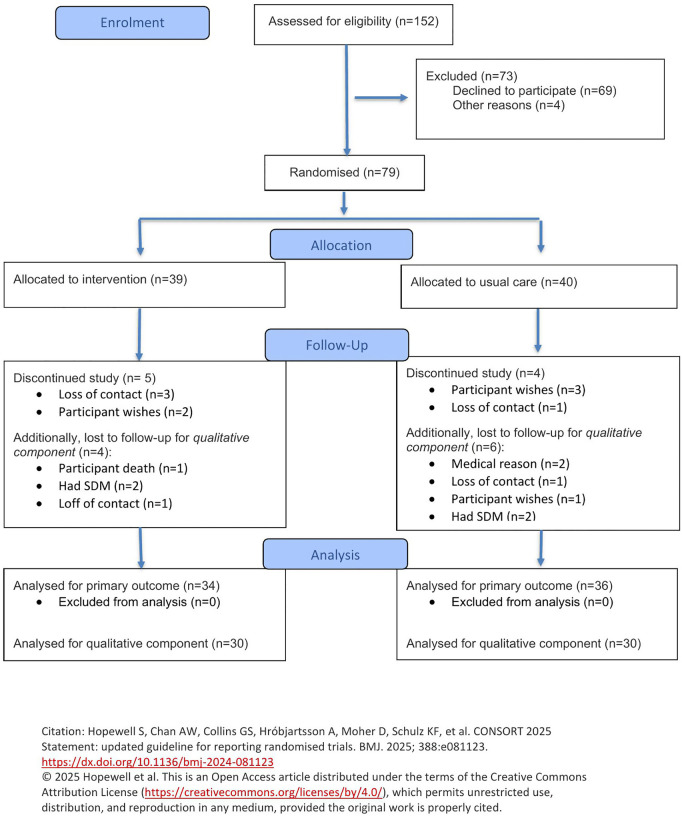
CONSORT patient flow diagram.

Informed consent was obtained from 79 participants with 6 SDMs consenting (Fig 2). Thirty-nine participants were randomized to the PN group, and 40 to the SOC group. Nine patients were lost to follow-up. Three-month follow-up data was collected from 70 participants (PN = 34, SOC = 36). Prior to discharge, two family caregivers withdrew from the study. Of the remaining 15 family caregivers, seven were caregivers of patients assigned to the SOC group and eight were caregivers of patients that had been assigned to the PN group. Semi-structured interviews were conducted with all 15 caregivers.

Descriptives for the PN and SOC groups are delineated in Table 1. UHU ranged from 0 to 90 for the SOC group with two patients that remained hospitalized after discharge from the OU for an average of 39 days, 4 patients admitted to hospital for other reasons for an average of 4.75 days, and 3 patients with unplanned ED visits. UHU ranged from 0 to 88 for the PN group with 13 patients remaining in hospital in another hospital bed for an average of 34.5 days following discharge from the OU, 0 patients had further hospital admissions and 2 patients with unplanned ED visits. In both groups, a large number of participants reported no UHUs. Therefore, the analysis was conducted with UHU as a binary variable with 0 representing no UHUs and 1 representing UHUs.

**Table 1:** Descriptives for standard of care and patient navigator groups.

	Quantitative Analysis (*N* = 70)				Patient Interviews (*N* = 60)				Caregiver Interviews (*N* = 15)			
	Standard of Care		Patient Navigator		Standard of Care		Patient Navigator		Standard of Care		Patient Navigator	
	*n*	%	*n*	%	*n*	%	*n*	%	*n*	%	*n*	%
**Sex**												
Female	30	83.3	27	79.4	25	83.3	23	76.7	6	85.7	8	100.0
Male	6	16.7	7	20.6	5	16.7	7	23.3	1	14.3	0	0.0
**Marital Status**												
Single^1^	18	50.0	16	47.1	14	46.7	13	43.3	2	28.6	2	25.0
Relationship^2^	18	50.0	18	52.9	16	53.3	17	56.7	5	71.4	6	75.0
**Ethnicity**												
Asian	1	2.8	0	0.0	1	3.3	0	0.0	0	0.0	1	12.5
Métis	0	0.0	1	2.9	0	0.0	1	3.3	0	0.0	0	0.0
White	35	97.2	33	97.1	29	96.7	29	96.7	7	100.0	7	87.5
**Education**												
≤ High School	16	44.4	17	50.0	3	10.0	8	26.7	2	28.6	2	25.0
> High School	20	55.6	17	50.0	27	90.0	22	73.3	5	71.4	6	75.0
**Age** Mean(*SD*^*3*^)	77.2(6.9)		77.0(8.4)		77.4(6.6)		76.4(8.1)		64.6(6.9)		61.3(10.1)	
**PFFS**^4^ Mean(*SD*^*3*^)	19.3(5.2)		20.5(4.5)		18.7(5.2)		19.8(5.2)					
**PS**^5^ Mean(*SD*^*3*^)	7.8(2.4)		8.4(2.1)		8.4(1.9)		8.6(1.6)					
**UHU**^6^ Mean(*SD*^*3*^)	18.1(26.7)		13.3(21.4)		16.5(27.1)		13.1(21.7)					

Note: ^1^ Single = single, widowed, separated/divorced; ^2^ Relationship = common-law partner, married; ^3^ SD = Standard deviation;

^4^PFFS = Pictorial Fit-Frail Scale-Acute; ^5^ PS = Patient satisfaction; ^6^ UHU = Unplanned healthcare utilization

### Quantitative

We originally intended to treat UHU as a count variable. However, UHU was highly zero-inflated with 0 UHU reported by 18 and 19 participants in the SOC and PN groups, respectively. In addition, there was dispersion issues with large variance to mean ratios (SoC = 711.70/18.11; PN = 456.69/13.26). Therefore, to address these issues and avoid the increased chance of Type I errors, biased coefficients, and incorrect inferences about predictors, we decided to conduct a model comparison and selection procedure. Model selection was guided by the Bayesian Information Criterion (BIC), with the model exhibiting the lowest BIC retained for interpretation [44,45].

All SEMs were conducted using *MPlus* (Version 8.10; Estimator = MLR). The initial SEM treated UHU as a count variable without adjustments for the large number of zeros or overdispersion (BIC = 2426.14). A zero-inflated Poisson model was then estimated to account for the excess zeros (BIC = 1231.13), and a negative binomial model was tested to address overdispersion (BIC = 751.69). The zero-inflated negative binomial analysis simultaneously addressed both issues (BIC = 739.60), while the hurdle model evaluated UHU as a binary variable in the first stage and as a count variable in the second stage (BIC = 739.84).

Because the count-based SEMs adjusting for zero inflation and/or overdispersion are more complex and require larger sample sizes for stable estimation, the adequacy of statistical power was uncertain. Therefore, a sixth SEM was estimated treating UHU as a binary outcome (BIC = 418.05). This model demonstrated the lowest BIC and was retained for interpretation (see Table 2).

**Table 2:** Parameter estimates, odds ratios, and confidence intervals for logit SEM with moderation.

Outcome Variable	Predictor Variable	Estimate	Standard Error	CI Lower 2.5%	CI Upper 2.5%	*z*	*p*
Patient Satisfaction	Group	0.150	0.112	−0.069	0.369	1.345	0.179
							
Healthcare Utilization	Group	−0.085	0.134	−0.347	0.177	−0.635	0.525
	PFFS2	−0.170	0.163	−0.490	0.149	−1.045	0.296
							
	Group*PFFS2	0.367	0.169	0.036	0.698	2.173	0.030
							
**Logistic Regression Odds Ratios**							
Healthcare Utilization	Group	0.724	0.371	0.265	1.979	–	–
	PFFS2	0.935	0.061	0.824	1.062	–	–
							
	Group*PFFS2	1.253	0.140	1.007	1.560	–	–

*Note: PFFS2 = Pictorial Fit-Frail Scale at time of consent.*

The a priori power analysis was based on the assumption of a count outcome, which typically demands a larger sample size to achieve comparable power relative to binary models due to greater variability and model complexity. Accordingly, the available sample was considered sufficient for analyses specifying a binary outcome. Although treating UHU as binary reduced information about the magnitude of UHU, this approach provided a more parsimonious and stable model given the sample size. We acknowledge this limitation and recommend that future research obtain larger sample sizes and retain the full count information. Given these considerations, the present findings should be regarded as hypothesis-generating rather than hypothesis-confirming.

As noted in Table 2, there was no statistically significant difference by intervention group (PN or SOC) with respect to PS or UHU. Additionally, the level of frailty did not significantly predict UHU. However, the interaction of the level of frailty and the intervention group did significantly predict UHU. Patients who were less frail in the SOC group had higher UHU compared to patients that were less frail in the PN group. Further, patients who were frailer in the SOC group had a lower number of UHU compared to patients that were frailer in the PN group.

### Qualitative

Thirty patients from the PN and SOC groups were interviewed (see Fig 2, detailing qualitative component specific loss to follow-up). Seven caregivers in the SOC group and eight in the PN group were also interviewed (see Table 1 for further descriptives). Predominant themes from the reflexive thematic analysis of the semi-structured interview data are described below and outlined with representative quotes in Tables 3 and 4.

**Table 3:** Comparison of themes for standard of care patient and patient navigator patient groups.

Standard of care Patients		Patient Navigator Patients	
Patients relied on family and friends	*“Ah, you know, I, I guess because my daughter always came and was there to help with different things, there was no problem, and, no, I felt everything was good that way.”* (1509SOC) *“Well, I had help. My, my husband was here, of course, and he, he definitely helped me. I don’t know, you know, I think there may be issues if you’re, you know, that was one of the questions or, you know, do you live alone or do you have help when you go home...and, uh, I had that help…so, you know, without that help, that would’ve probably been a little different, you know.” (2140SOC)*	Patients found Patient Navigators supportive and helped with care	*“Oh, well, I think this with [PN] to know, to have someone that comes into the room and checks on you and, and he is focused about your needs and what you need when you go home was a plus. Like I say, neither one of us knew what the heck we were doing and so it helps.” (1025PN)* *“Well, you knew that it, he was there…and if anything had gone wrong, I would’ve called him in a minute…and I knew that he’d go the extra mile for me, I could just tell that.” (1015PN)*
Poorly coordinated discharges negatively affected patients and their families	*“Well, actually, they gave me a piece, a paper, but no one really filled it out…But all they told me was I, I had to go see my doctor within a certain amount of time to get the staples out, that’s all. But nothing about any kind of exercises or anything like that, really…yeah, just, just to get me out of the hospital…but it was really, like, no, no good information.”* (1216SOC) *“…when I got home, I had to, neighbours helped me get into the house and it took us about 20 minutes to get me into the house. They sort of lifted me up…to get in, erm, rather than crawl up the stairs because I couldn’t…well, if they had assessed me well and then they would have realised that I couldn’t get into the house…by myself and that I should’ve had a, erm, an ambulance service to, with a stretcher bring me into the house sort of thing.” (1403SOC)*	Patients relied on family and friends for support and care	*“Yeah, I think, my, my boys were so good about being there early and got me the wheelchair, and all that stuff. And getting all my stuff together. I mean, if somebody didn’t have family, that might be, you might need some more supports upon discharge. Right? If someone was just going to pick you up at the front door or something.”* (1029PN) *“Um, well my daughter, my daughter had done most of it, she had, um, there was… Somebody came to the house and told her what, probably what I would need, um, as far as, as far as the layout of the house…so, they said, they said, well you’ll need some grab bars here, and maybe some there. So, she went and had them all put in. And, uh, that was before I got home.” (1002PN)*
Support, care, and communication facilitated smooth transitions home	*“Um, well, you know, I come right home, and then I had Extra-Mural, and I had workers here at the house. I had them in the morning and then to get supper for me…and, and, ah, the occupational therapist was here, and all. It was just, it was just wonderful…when I came home, everything was already done…I didn’t have to do anything when I got home…um, you know, they, they just help so much, and they brought me a bedrail so I could… because at the time, I couldn’t get up or anything by myself…and they brought a bedrail for me, and, oh, they were just so good…yeah, I had a, a really, really good experience. You know, not with my broken hip but…you know, I, I don’t think anybody could be treated any better.”* (1129SOC) *“When I came home, ‘cos I had Extra-Mural, ah, stopping in here…and, um, because of my age, I was given homecare workers for a while, and, ah, no, I found Extra-Mural came and, and checked me on my exercises, and, ah, ah, and checked me on different things, came and checked my blood pressure, made sure it was working, and, no, it was lovely that way.” (1509SOC)*	Patients did not always need Patient Navigator support	*“…basically no, because I had Extra-Mural come in and, I mean, everything was, kind of, provided for me…and when I first come home from the hospital my son was here, he stayed for a month, and my daughter-in-law, so I had lots of help around the home.” (1020PN)* *“I mean, for someone that probably wasn’t, um, used to service…I’m, I’m sure she would’ve been extremely helpful…uh, in my situation, probably not needed…but, you know, I mean, there’s, there’s such a vacuum when you leave the hospital…that I did appreciate somebody checking in…it, because I didn’t run into any problems” (1008PN)*

**Table 4:** Comparison of themes for standard of care family caregiver and patient navigator family caregiver groups.

Standard of Care Family Caregivers		Patient Navigator Family Caregivers	
Patients relied on support from family and friends throughout the care journey	“*Yeah, I do. I mean, because I’m a [healthcare provider], I do what I can when I’m there with her…if I’m there after supper, then I would get her washed up, and get her pyjamas on, and get her ready for, for the nighttime. Um, I’ve brought her in new food, you know, meals. Um, actually, just yesterday um, we took her out of the nursing home, and we went to Swiss Chalet with her for supper. So, it’s, you know, the nursing home gives her, her medication and all those kinds of things, so it’s basically just getting her dressed, or undressed. Sort of thing when I’m there*.” (1927C)	Caregivers found PNs supportive and helped with care	*“Uh, no. I just, I just felt [PN] was great. And he did help me and, and our family. And I’m glad I decided to do the whatever it was called, the research. Or the program. I forget what it was called. But…’cause it, it did, it did really help us.”* (1028C)
Ongoing stress for caregivers throughout the care journey	*“Overwhelmed [laughs]…well, I mean, there’s, there’s a lot to deal with, right? And decisions that need to be made. And, um, yeah, it’s, there’s a lot to do, right?”* (1110C)	Patients relied on support from family and friends throughout the care journey	*“Yes. I got prepared [for the transfer back to home]. For that. I got the lift, like I said. And there was family in place. And we were looking after her in the hospital anyway. So, it was easier for everybody to look after her at home. And we got her a hospital bed, a lift chair, and a Sara Stedy. I mean, if we weren’t able to afford that type of thing, she wouldn’t have been discharged when she was. ‘Cause we wouldn’t have been able to provide the care that she needed to get up to go to the bathroom and that kinda thing*.” (1028C)
Caregivers with previous knowledge about healthcare system found it easier to navigate	*“Oh, yeah, yeah, everything [communication in the hospital] was really good. I worked in health…So, I, I pretty, er, I know how the system works. And I, er, I had no problem getting information, because a lot of the times I knew all I had to do was ask her nurse. If I needed information about any, anything, and they’re always more helpful. Yeah. They’d find out anything I ask, er, really quickly, yeah. Any concern or any concerns I had about her, er, they’d answer right away, yeah. To my satisfaction. So, yeah, the whole hospital process was, er, I found really good. Really, really good. And the whole process going right from the start to the finish.”* (1708C)	Ongoing caregiver stress throughout care journey	“... *my biggest worry is if she turns or moves to quick, she’s gonna go down. But there’s no telling her that, and so far, she’s been home two or three weeks, and she’s doing fine. So, one day at a time. That’s, that’s how it. That’s how we have to accept it. Otherwise, you know, I could worry about her all the time. But I’m trying real hard not to. Now, if something happens, I don’t know how I’m gonna feel*.” (2032C1)

### Patient navigator group patient themes

#### Patients found PNs supportive and helpful with care.

Most patients found their PNs supportive and helpful with their care. While on the OU, PNs provided information to patients regarding their care by seeking answers to their questions from the chart or from HCPs. They regularly checked in on participants in the hospital and post-discharge, which for some provided social and emotional support and a sense of reassurance. PNs helped acquire the equipment needed prior to patients being discharged. They also provided information on available community resources and assisted the patients, ensuring that necessary follow-up care was received.

#### Patients relied on family and friends for support and care.

Throughout their care journey, many patients relied heavily on friends and family. Family/friends helped to ensure everything was in place post-discharge prior to their arrival by acquiring equipment, organizing community care, and coordinating the patient’s transportation. They continued to provide care and support to participants once they arrived at their post-discharge location.

#### Patients did not always need PN support.

Although most patients found that PNs were supportive and helpful, several participants were not as reliant on their support since they already had the equipment, care, information, and/or resources to support their recovery. Patients who had undergone previous surgery often already had equipment, and others had supportive services in place prior to discharge. Some mentioned not needing additional help because they had support from family/friends or their family physician. A few participants were not as reliant on their PNs because they did not have any health or care related concerns. Despite being appreciative of the follow up, these participants felt they were already well-informed of the resources available and did not require additional support.

### Standard of care group patient themes

#### Patients relied on family and friends.

Most SOC patients found family/friends vital sources of support throughout their care journey. During their hospital stay, family/friends provided patients with a range of support, including physical and moral support and advocating for their care. Family/friends supported transitions in care and post-discharge needs by arranging care, equipment, and transportation. Many patients expressed gratitude for support from family/friends, and some raised concerns for others who did not have that type of help.

#### Poorly coordinated discharges negatively affected patients and their families.

Many patients discussed poorly coordinated discharges which negatively impacted their care experiences post-discharge. The most common issues were that the discharge felt rushed and poorly communicated. Patients often mentioned the OU was short-staffed. Several patients felt that they were discharged without receiving adequate information about their surgery, discharge time or location, post discharge instructions, and supports for care needed at home. Being discharged home without pre-arranged care and/or necessary equipment negatively impacted some patients’ transitions and well-being. Without the pre-arrangement of care, several participants said they struggled physically and mentally with the transition home. Some struggled with navigating the healthcare system on their own (e.g., not knowing how to set up physiotherapy post-discharge).

#### Support, care, and communication facilitated smooth transitions home.

Many patients discussed that the most pivotal factor facilitating smooth transitions out of the hospital was having support and assistance from family/friends. Patients also discussed the importance of having care and equipment arranged, either by the hospital or family caregivers, prior to arriving home. Most participants felt prepared to go home when the necessary care and equipment were in place. More than half of participants discussed the benefits of support from community care services (such as personal support workers) in their transition home. Also, clear and useful discharge information was a major facilitator for smooth transitions post-discharge.

### Patient navigator group caregiver themes

#### Caregivers found PNs supportive and helped with care.

All caregivers found PNs supportive and helpful throughout the transition process. PN support and availability not only strengthened communication with families, but also provided a sense of reassurance for caregivers, knowing they could contact the PN with questions or concerns. A few caregivers mentioned specifically that the PN alleviated stress when helping navigate the healthcare system. Some caregivers described the PN as emotionally supportive and understanding, listening to their concerns, and providing valuable advice.

#### Patients relied on support from family and friends throughout the care journey.

Caregivers described how patients often relied on support from family/friends throughout their care journey. Family/friends were instrumental in helping patients with transitions in terms of planning and preparations, including transportation as well as purchasing and setting up equipment. Once discharged from the OU, patients relied on family/friends for help with care, household tasks, and equipment needs.

#### Ongoing caregiver stress throughout the care journey.

Caregivers experienced ongoing stress throughout the care journey. Many caregivers expressed concern and worry about future falls. Only one caregiver in the PN group mentioned being more tired than before.

### Standard of care group caregiver themes

#### Patients relied on support from family and friends throughout the care journey.

Caregivers described how patients often relied on support from family/friends throughout their care journey. Most caregivers visited and assisted with the patient’s care while they were in the hospital, including advocating for patients and facilitating communication with HCPs. They were instrumental in assisting patients with the transition from the OU (e.g., arranging transportation/ equipment). Patients relied on family/friends for assistance with various daily activities and emotional support after discharge. Some caregivers noted that without their support, the patient would have encountered significant difficulties or would not have been able to return home.

#### Ongoing stress for caregivers throughout the care journey.

In discussing their experiences both pre- and post-fracture, it was evident that most caregivers had ongoing stress throughout the care journey. Caregivers expressed concern about future falls and described periods of being emotionally overwhelmed with the many decisions, life changes, and issues they navigated. In a few cases, caregivers felt that they were on their own to navigate aspects of the system, leaving them feeling blindsided and lost at times. Some caregivers mentioned caregiver burnout and struggled to find time to care for themselves amid caring for others.

#### Caregivers with previous knowledge about the healthcare system found it easier to navigate.

A few caregivers had previous knowledge about the healthcare system (e.g., working within healthcare, patient previously admitted with a fracture) and thus found the process easier to navigate. For instance, caregivers felt they knew what to expect, had familiarity with system processes, and had no problems getting the information they needed along the way.

## Discussion

Quantitative findings regarding the impact of PNs on UHU revealed a significant interaction effect. Specifically, the SOC patient group had more UHU’s at lower levels of frailty, whereas the PN patient group t had fewer UHUs at lower frailty levels but more UHUs as frailty increased. There are three possible interpretations for this pattern. First, this finding may suggest that PNs facilitated more appropriate use of healthcare resources, aligning with and possibly reflecting the qualitative findings regarding the provision of healthcare information and connecting patients with resources by PNs. Second, it is possible that the PNs failed to prevent deterioration among patients as frailty increased. Third, this finding may be spurious due to sample size limitations, despite having met the a priori power analysis recommendations. Further research is needed to clarify these possibilities and to explore the mechanisms underlying the relationship between PN involvement and UHU across frailty levels, as there has been previous research that found patient education, follow-up, and PNs have the capacity to reduce patient readmission to hospital [10,11,22,23].

Although quantitative results did not find the outcome of overall PS with healthcare was impacted by PNs, contrary to some research in a different patient population [24], the qualitative results found high patient satisfaction expressed with PN care. Comparison between the thematic analyses of SOC and PN patients found that while there was some consistency between groups (e.g., relying on family) the PN group had unique themes, detailing the positive impact of the PN, particularly in relation to the provision of information and support. Patients found that PNs were helpful throughout the entire care trajectory and contributed to the overall well-being of participants and their families. These qualitative findings highlight the intervention’s success regarding the primary goal of patient navigation: to guide patients through complex healthcare continuums and eliminate barriers to timely care [14]. Using a single question measure for PS appears to have been a limitation, as it did not capture the positive impact of PNs detailed in the qualitative results.

Although the results of the qualitative component found most patients indicated that PNs were helpful, some patients felt that the support was not as necessary. These patients generally had fewer care needs and more family support. This reflects the quantitative findings whereby less frail patients within the PN group had less UHU. These findings suggest that PN services may be more appropriate for certain groups of individuals and align with suggestions that patients with complex needs (e.g., increasing frailty) may benefit most from PNs [16].

Support from PNs may be helpful in reducing caregiver burden, as the qualitative component found SOC patients relied further on family/friends. Notably, the SOC group lacked information and support that a PN could have provided. Findings from the qualitative component found the perspectives of caregivers were consistent with those of patients. Specifically, SOC group caregivers discussed patients relying on support from family/friends throughout their care journey, whereas caregivers in the PN group predominantly discussed finding PNs supportive and helpful. Both caregiver groups discussed the ongoing stress that they felt throughout the care journey; however, for the PN group this topic was less prevalent. These findings point to the helpfulness of PNs for not only patients, but their caregivers as well.

### Study limitations and directions for future research

This study has several limitations that should be considered. First, its generalizability is limited, as it was conducted at a single hospital site with participants who were primarily White and female. Second, limitations related to the dependent variables warrant caution in interpretation. PS was measured using a single-item question to reduce participant burden and improve follow-up rates at the 3-month follow-up; however, this approach may have limited the ability to capture the full complexity of PS. Similarly, the high proportion of zeros in UHU required model comparison and selection procedures that ultimately led us to treat UHU as a binary variable, thereby reducing the amount of information captured. In addition, UHU was self-reported, introducing the potential for self report bias. Consequently, these findings should be considered hypothesis-generating rather than confirmatory. The heterogeneity of patient fractures within the study is also a limitation, as the results are then non-specific regarding differentiation of the complexity of various types of geriatric fractures. Future research should aim to enhance generalizability through multisite recruitment, consider longer recruitment periods, and ensure measures and sample sizes allow for capturing the full range of outcomes.

## Conclusions

The evidence for the effectiveness of PN programs are scarce, particularly outside the USA and cancer care programs [46]. This study extends the literature by providing a better understanding of the positive impacts a PN can have on older adult inpatient care and transitions in care experiences, as found in the qualitative component. Both patients and caregivers found PNs supportive and helpful with care. PNs were shown to be particularly helpful for participants with higher care needs and would also be beneficial for those patients with fewer family supports. The ambiguity of the quantitative results points to the need for further research on the effectiveness of PN programs, particularly regarding healthcare usage.

## Supporting information

S1 File CONSORT 2025 checklist. (PDF)

S2 File Study protocol. (PDF)
