# Supplementary material for: Investigating patient navigator impact on older adults’ transitions from acute care: A randomized controlled trial with embedded qualitative component
Source: PLoS One. 2026 Feb 4;21(2):e0341251. doi: 10.1371/journal.pone.0341251 (PMC12871977; doi:10.1371/journal.pone.0341251)
Supplement: S2 File — (PDF) [file pone.0341251.s002.pdf]

The CHARM Study-
Coordinating transitions from hospital for older adults with fractures: An interventional mixed methods study

Principle Investigator

Ian Watson, BSc, MHSc, Administrative Director, Trauma NB

Co-Investigators

Pamela Jarrett, MD, FRCPC, FACP, Associate Professor of Medicine, Dalhousie Medicine New Brunswick, Geriatrician, Horizon Health Network

Natasha Hanson, PhD, Research Manager, Research Services, Horizon Health Network

Tracy Freeze, PhD, Research Methodologist, Research Services, Horizon Health Network

Karla Faig, BScPT, Research Manager, Collaborative Care Seniors Health at Horizon Health Network

Leanne Skerry, MA, Research Coordinator, Research Services, Horizon Health Network

Morgan Nesbitt, MA, Research Assistant, Research Services, Horizon Health Network

Susan Benjamin, RN, BN, Trauma Resource Nurse, Trauma NB

Julie Ringuette, RN, BScN, Trauma Coordinator, Trauma NB

Leisa Ouellet, RN, BN, Trauma Coordinator, Trauma NB

Tushar Pishe, BSc, MD, CCFP(EM) FCFP, Medical Director, Trauma NB

James Wagg, MD, FRCSC, Orthopaedic Surgeon, Horizon Health Network

Richard Louis, MD, MHA, Injury Prevention Specialist, Trauma NB

Shelley Doucet, PhD, Professor, Nursing and Health Sciences, University of New Brunswick, Saint John

Alison Luke, PhD, Research Associate, Nursing and Health Sciences, University of New Brunswick, Saint John

PROTOCOL SUMMARY

Background: Fall-related injuries like fractures are on the rise among older adults in New Brunswick. These injuries can lead to hospitalization and adverse health effects. Moreover, transitions from acute care can be complicated and overwhelming, especially for patients and their families. Researching patient navigators as a means of enhancing inpatient care, while also ensuring successful transitions in care for patients, may have positive impacts and help older adults successfully age in place.

Objective: This study seeks to investigate the effects of having support from patient navigators in helping older adults admitted to the Orthopedic Unit with a fracture. Specifically, the objectives are to investigate whether there are differences between patients with patient navigators and those patients receiving standard of care, regarding: the length of stay in acute care; healthcare utilization post-discharge; patient and family experience and satisfaction with care; and, healthcare provider experiences working with patient navigators.

Methods: This study uses a mixed-method concurrent embedded design, in which the quantitative randomized control trial has an embedded qualitative component.

Potential Benefits/Risks: This study is considered low risk. Potential benefits of this study include a better understanding of the impact of support from a patient navigator on inpatient care and patient transitions. This information will be used to inform the development of practical recommendations for policymakers and clinicians on how to enhance inpatient acute care and successful transitions for older adults.

The CHARM Study
Coordinating transitions from hospital for older adults with fractures:
An interventional mixed methods study

Background

With Canada's aging population[1], it is crucial that health and social services are responsive to the needs of older adults. This is particularly true in New Brunswick (NB) where the number of older adults is growing quickly[1]. Transitions in care for older adults are a critical junction in their healthcare pathway, where there is the greatest vulnerability to disparities in health outcomes[2-4]. The transitional process is complicated by the multitude of activities surrounding the patient's discharge, such as the activation of varying hospital and community resources to facilitate the transition[5,6]. Supporting older adults' transitions out of hospital can place significant pressure on healthcare teams in hospital who are responsible for identifying needs and coordinating care to any given location upon discharge. This is especially true for hospital-based healthcare providers who work predominantly with frail seniors who often have underlying complex medical, cognitive, functional, and social needs in addition to acute issues such as fractures. Findings from a recently completed study by some of the research team members indicated that many patients and families found that transitioning from hospital to home was an impenetrable and daunting process; thus, guidance and open communication is needed to facilitate the transition[7,8]. Fortunately, research suggests that patient education and follow-up can be effective means of reducing patient readmissions[9,10] .

Patient navigators are a patient-centred way of providing this education and follow up, as well as coordinating care and discharge[11] . The main goal of patient navigators is to guide patients through complicated healthcare systems, and to improve health outcomes and access to timely care by reducing socio-economic, informational, and logistical barriers[11-13]. Some tasks through which this is accomplished include: disease- and health system-related education; communication and coordination between care providers; arranging appointments and referrals; and, strengthening the interpersonal relationship between the patient and care team[14,15]. Patient navigators help the patient identify and access appropriate programs, services, or resources; thus, ensuring the transition to community is as seamless as possible.

Patient populations who may benefit most from patient navigators are those with complex needs; for instance, patients who make frequent visits to the hospital, are likely to be readmitted, or who are negatively impacted by social determinants of health[16]. Patient navigation programs are most often employed in the context of cancer or other chronic illnesses in which healthcare delivery may be fragmented between different providers and healthcare settings[11]. As such, patient navigation may be particularly important for frail older adults who may have multiple healthcare providers in different settings, among which there may exist discontinuity in care and poor

communication[17,18]. This applies to older adults who have recently been hospitalized with such events as a recent fracture. Interventions to improve care coordination and communication between healthcare providers, both in the inpatient setting and after discharge, may be effective in reducing readmissions and length of stay[19,20] and enabling successful aging in place. Researchers have found that implementing patient navigators for patients at risk of readmission resulted in decreased readmission for participants aged 60 or older[21,22], and may also increase satisfaction with care[23]. As key components of patient navigation, patient education and follow-up may be effective in reducing patient readmissions[9,10]. The patient-centred approach of patient navigators can support patients and families in accessing necessary resources and services during their transitions in care; thereby, facilitating them in a timely manner and fostering self-management[14,24].

Patient navigation may be instrumental in provinces with older populations. New Brunswick (NB) has one of the highest proportions of older adults over the age of 65 years across Canada. Fall-related injuries among individuals in this age group are a leading cause of hospitalization, with approximatively 30% of older adults experiencing one or more falls each year[25]. Research has also suggested that falls are the direct cause of 95% of all hip fractures[25]. Each year in NB, hip fractures result in over 500 hospitalizations with an average length of stay of 22.5 days and account for over 11,000 bed days every year[26]. In addition, many older adults who fall experience a severe increase in frailty and never regain their independence. It has been estimated that 40% of the elderly who are injured in a fall will require nursing home placement[25].

Frailty is a medical condition that is more likely to develop in older adults, and can result in reduced health and function[27]. A person with frailty does not have the ability to cope with minor illnesses and these stressors may trigger deterioration[28,29]. It is postulated that approximately 24% of older adults are frail and it is this group of older adults that are at the highest risk of adverse outcomes from fractures[30-32]. While increasing frailty can result in worse health and social outcomes[33], there is also an opportunity to provide proactive management and prevention to allow some of these frail older adults to return home and successfully manage in place with appropriate supports.

Following an examination of provincial hospitalization data estimates, Trauma NB has identified a significant upward trend in estimates of falls-related injury in older adults over the next 20 years (see Figure 1). These fall-related injuries, such as fractures, often lead to adverse health outcomes for frail older adults including admission to nursing or special care homes and even death[30,31,34]. For those older adults who fall and are successfully treated in hospital, one of the main goals should be to support them as they return to the community so that they can successfully age in place as long as possible.

Present Study

In recognition of the importance of these issues, a strategic priority for Trauma NB is to enhance inpatient care for older adults and their families. This priority also aligns with Horizon Health's recently completed strategic plan, which includes the pillars of "Efficient and appropriate Care," as well as, "A system that is wrapped around the patient/clients' needs"[35]. These pillars both connect to the need for accessibility of care, particularly for people as they age, as stated by the Government of New Brunswick in a recent discussion paper[36]. Researching patient navigators as a means of enhancing inpatient care, while also ensuring successful transitions in care for patients, is a strategic focus for Trauma NB. This topic was also discussed by stakeholders in building Horizon Health's strategic plan, where it was directly mentioned that there is a need to "Help patients navigate the system" and ensure that "Inpatient length of stay is minimized"[35]. Together with Drs. Doucet and Luke, Trauma NB team members are currently finishing a scoping review of the characteristics and impact of hospital-based patient navigation programs to support injury-related trauma patients and their caregivers, as well as a pan-Canadian environmental scan of patient navigator programs for this population. Members of the current research team are also working to complete an ongoing study on isolated hip fractures in New Brunswick (including Ian Watson, Dr. Jarrett, and Susan Benjamin), in order to understand the full scope of health outcomes with this type of fracture. Also, previous research by team members Drs. Jarrett and Hanson found many areas for improvement in the transition from hospital to home for frail older adults[7]. The current proposed project seeks to build from this considerable previous research, addressing some of these important issues raised by piloting the implementation of patient navigators for older adults admitted with fractures to facilitate and support their transitional care needs. This initial pilot will be provided in English, with future plans for bilingual implementation, depending on the research results.

The overall goal of this project is to investigate effects of the implementation of patient navigators, working alongside the usual healthcare team on Orthopedic Units targeted to assisting adults aged 65 and older admitted to an Orthopedic Unit in one hospital in New Brunswick with a fracture, as compared to those patients who receive the usual standard of care. Specifically, the project objectives are to investigate whether there are differences between patients with patient navigators and those patients receiving standard of care, regarding: the length of stay in acute care; healthcare utilization post discharge; patient and family experience and satisfaction with care; and, healthcare provider experiences working with patient navigators.

This project will include consideration of level of frailty and seeks to understand if a patient navigator approach to care can allow these older adults to have improved health outcomes and continuity of care when transferred from acute care.

Research Questions

Research questions one through four and their corresponding hypotheses (H1-H4) will be assessed in one structural equation model (see Figure 2). Research questions five through seven and their corresponding hypotheses (H5-H7) will be assessed in a second structural equation model (see Figure 3). Research questions eight and nine will be answered with qualitative research; thus, results are not hypothesized.

RQ1. Does support from a patient navigator increase positive patient experiences of their acute care hospitalization compared to standard of care?

RQ2. Does support from a patient navigator increase positive family experiences of their loved one's acute care hospitalization?

RQ3. Does support from a patient navigator reduce length of stay in acute care?

RQ4. Does the support from a patient navigator weaken the relationship between frailty and length of stay in acute care?

RQ5. Does support from a patient navigator increase patient satisfaction with care during the three-month follow-up period?

RQ6. Does support from a patient navigator reduce unplanned healthcare utilization during the three-month follow-up period?

RQ7. Does support from a patient navigator weaken the relationship between frailty and healthcare utilization?

RQ8. What are the experiences of healthcare providers in working with the patient navigators or working as a patient navigator?

RQ9. What are the experiences of patients and family caregivers receiving the support of a patient navigator, as compared to those who did not?

Hypotheses

H1. Patients receiving the support of a patient navigator during acute care will report higher levels of positive experiences compared to patients receiving the standard of care.

H2. Families of patients receiving the support of a patient navigator during acute care will report higher levels of positive experiences compared to families of patients receiving the standard of care.

H3. Patients receiving the support of a patient navigator during acute care will spend fewer nights in acute care.

H4. Patients with greater levels of frailty will spend more nights in acute care, but the support of a patient navigator will weaken that relationship.

H5. Patients receiving the support of a patient navigator will have higher levels of self-reported patient satisfaction with the healthcare they received during follow-up than patients receiving the standard of care.

H6. Patients receiving the support of a patient navigator during the three months post-acute care will have a lower level of unplanned healthcare utilization when compared to patients receiving the standard of care.

H7. Patients with higher levels of frailty will have higher levels of unplanned healthcare utilization during the three months post-acute care, but the support of a patient navigator will weaken that relationship.

Methods

Participants

Patients 65 years of age and older admitted to the Orthopedic Unit at SJRH (OU) for a fracture, who are English-speaking, will be included in this study. They or their substitute decision makers (SDM) will be approached by non-study related personnel, within the patient's circle of care, to see if they are interested in participating in the study. If they are interested this will be relayed to the research team, who will contact the patient/SDM with project information and provide them with the Patient Consent Form. Informed consent will be obtained prior to the collection of data and any other research procedures. In cases where the SDM has been asked to make medical decisions for the patient, informed consent for the patient's participation will be obtained from the SDM, and assent will be obtained from the patient. These participants who lack capacity will be included in this study given that: 1) The research question can be addressed only with participants within the identified group; and, 2) The research entails only minimal risk, and has the prospect of providing benefits to a group that is the focus of the research and to which the participants belong.

A series of simulations to estimate the sample size needed to observe the patient navigator effect on patient experiences (PE), family experiences (FE), and length of stay (LOS) at the time of discharge from acute care hospitalization were conducted in MPlus. Another series of simulations were conducted to estimate the sample size needed to observe the patient navigator effect on unplanned healthcare utilization (HU) and patient satisfaction (PS) of healthcare during the three months following acute care hospitalization discharge. Models of these relationships are illustrated in Figures 2 and 3, respectively. Comparable studies reporting quantitative data and effect sizes were lacking; thus, moderately conservative effect sizes were simulated. With the exceptions of the relationship between frailty and HU/LOS (d=.5)[30-33], a small to medium

effect size (*d* = 0.35) was used for simulations of these primary relationships. A total sample size of 60 patients or 30 patients per group will be necessary to obtain an approximate power of approximately .8 and alpha of .05 for H1-H3 and H5-H6, though a larger sample size is preferable to ensure stability of the model during estimation and to reduce the chance of Type II error for the underpowered H4 and H7. There is an average of 18 patients per month admitted with a fracture from the SJRH Emergency Department to the OU. Therefore, if patients were recruited to the study for a period of six months, sample size requirements should be met or exceeded assuming a 60% recruitment rate.

Patients who agree to participate will identify a family caregiver if they would like one to be approached to participate in the research study. Family caregivers will be asked by a research team member if they would also like to participate in the study and provided with the Family Caregiver consent form. Family caregivers of patients who agree to participate in the research will be asked if they would also like to participate. Therefore, it is expected that there will be a similar number of family caregiver participants as patient participants. Informed consent will be obtained prior to the collection of data and any other research procedures.

HCP who worked with the patient navigators and the patient navigators themselves will be approached, after all patient participants have been discharged from the OU, about participating in a semi-structured interview at their convenience with a member of the research team (the patient navigators will not be conducting any of these interviews or recruiting HCP). HCP will self-refer to participate in the project. Posters will be put in HCP areas of the OU unit and snowball methodology will also be used, asking HCP to forward research team contact information to those other HCP they think may be interested in participating. It is expected that 10 HCP will be recruited to participate, which should provide sufficient saturation. HCP will be provided with the Healthcare Provider consent form to review. Informed consent will be obtained prior to the collection of data.

Measures

Demographic information

Demographic information will be collected from patients and family caregivers. Demographic information collected from patients will include: age at time of admission; gender; marital status; ethnicity; and, education. The following demographic information will be collected from family caregivers: age; gender; marital status; ethnicity; education; and, relationship to the patient (see Appendix A).

Patient and hospital stay information

The following patient information will be obtained from the patient's medical record concerning the latest hospital admission: date of hospital admission; reason for hospital admission;

treatment; diagnoses; medications; discharge dates (from acute care unit and from hospital); locations post-discharge and length; and, documentation associated with care transitions (including PN care plan), also including consultations placed to community agencies by hospital staff and discharge instructions. The information gathered for the Comprehensive Geriatric Assessment (see Appendix A) will also be used descriptively.

LOS is operationalized as the number of nights spent on the OU at the SJRH. HU is operationalized as the number of unplanned healthcare utilizations during the three months following discharge or transfer from the OU. Four items will be counted: length of stay in hospital following transfer from acute care (#nights); hospital emergency department visits (#times); and, total nights spent in hospital following initial hospital discharge (#nights). Other forms of healthcare utilization will be captured. However, they will only be subject to descriptive statistics, such as appointments with healthcare providers and use of programs such as Extra-Mural Program (EMP).

Pictorial Fit-Frail Scale (PFFS; Theou et al., 2019[37])

The PFFS is a practical, picture-based assessment of frailty in older adults[37]. The PFFS assesses 14 categories ranging from mood to bladder control. Each category is represented by a series of pictures corresponding to levels of impairment. Total scores can range from 0-43 with 43 representing severe frailty and 0 representing no frailty. The PFFS has been shown to have good test-retest and inter-rater reliability between nurses and geriatricians[38,39], content validity[37], and concurrent validity[40]. This is a proprietary scale and so has not been included in an appendix.

There are several versions of the PFFS allowing for use by either clinician or layperson and measurements of current fitness-frailty, usual-state fitness-frailty, or both. Because this research requires both current and usual-state fitness-frailty measurement, we will use the PFFS-Acute version. PFFS scores will be used both as a descriptive variable and a study variable. Following informed consent, the PFFS will be used to assess current frailty and usual frailty (operationalized as frailty prior to the patients' present hospitalization) of patient-participants. PFFS at discharge and PFFS at the three-month follow-up will also be recorded. The usual frailty total score will be used as a predictor of LOS (see Figure 2) and the initial current frailty total score will be used as a predictor of HU at follow-up (see Figure 3). A descriptive analysis of these four frailty scores (i.e., usual frailty prior to present hospitalization, current frailty assessed at the time of consent, current frailty assessed at the time of discharge from OU, and current frailty at follow-up) will be conducted.

Canadian Patient Experiences Survey Inpatient Care (CPES-IC)

The CPES-IC is a widely used standardized questionnaire developed by the Canadian Institute for Health Information[41,42]. It consists of 41 questions that assess the patient experience in acute care settings; thus, providing hospitals with important information regarding patient-centered care. The following sub-measures will be used as observed indicators of the latent variable Patient Experience: discharge management; internal coordination of care; emotional support;

communication with nurses; communication with doctors; overall hospital experience; and, involvement in decision-making.

CANHELP Lite Family Caregiver Questionnaire (CANHELP-L-CQ)

The CANHELP-L-CQ is a 23-item validated questionnaire assessing the family caregiver experience with the care their relative received while in hospital[43,44]. Though the original CANHELP questionnaire was developed for the family caregivers of patients with critical and/or life-threatening illness, the CANHELP Lite is used for assessing the satisfaction with the quality of care needed for the evaluation of programs or interventions. The following sub-measures will be used as observed indicators of the latent variable Family Experience: overall satisfaction; communication with doctors; characteristics of doctors and nurses; illness management; communication and decision-making; and, family caregiver involvement. This questionnaire is available in both French and English.

Global Patient Satisfaction Question

To measure patient satisfaction at the end of the three-month follow-up, one global question will be asked to patients: "Thinking about all of your healthcare experiences related to your fracture in the last 3 months, to what degree are you satisfied or dissatisfied with the services you have received? Use any number from 0 to 10, with 0 representing the least possible satisfaction with your healthcare experiences and 10 representing the most possible satisfaction with your healthcare experiences." This question was adapted from the global hospital rating in the CPES-IC.

Semi-structured Interview Guide for Healthcare providers

Healthcare providers who were involved in the care of patients assigned to a patient navigator will be asked about their experiences in dealing with the patient navigators, what they thought about the role of the patient navigator, and any improvements that could be made to the role (see Appendix D for HCP Interview Guide). Interviews will be conducted over the telephone or in person and audio recorded.

Semi-structured Interview Guide for Patients and/or Family Caregivers

Patients and/or family caregivers in both the intervention and SOC groups will be asked about their experiences in transitioning from acute care. These semi-structured interviews will take place at 3 months post-discharge from acute care. Those participants in the intervention group will be asked about their experiences with the patient navigator. All participants will be asked about any improvements that could be made regarding transitional care (see Appendix D for Patient and Family Caregiver Interview Guides). The decision as to whether to participate in these semi-structured interviews one-on-one or together (i.e., patient and family caregiver) will be left to patients and family caregivers to make. The interviews will be conducted in person or via telephone and audio recorded.

Procedure

Patients or their substitute decision makers (SDM) will be approached by non-study related personnel, within the patient's circle of care, to see if they are interested in participating in the study. If they are interested, this information will be relayed to the research team, who will contact the patient/SDM with project information and provide them with the Patient Consent Form (Appendix B). Patients who agree to participate will identify a family caregiver if they would like one to be approached to participate in the research study. Family caregivers will be asked by a research team member if they would also like to participate in the study and provided with the Family Caregiver consent form (see Appendix B). Informed consent will be obtained by a research team member before any data collection begins. Once signed to the study, patients will be randomly assigned to treatment condition (Patient Navigator or Standard of Care), and patient descriptives and the PFFS (usual and current state) will be recorded. A trained Research Assistant will be assigned to collect the data from the SOC group and help the Patient Navigator with data collection as needed. A notification letter of the enrolment of the patient in the research study will be sent to their primary healthcare provider (see Appendix E), in order to ensure they are aware of the patient's participation.

The patient navigator (PN), for those patients assigned to them in randomization, will initially conduct a Comprehensive Geriatric Assessment (CGA) in order to formulate personalized goals for each patient. The CGA entails gathering information regarding medical, functional, psychological, and, social assessments from the patient and/or family members and healthcare team members, when relevant. The CGA will allow for the development of a care plan needed for a successful discharge, and will be patient specific, attainable, and will focus on the following areas: Patient and family engagement, education and collaboration; Healthcare needs identified that need ongoing care and follow up after discharge; Pre-existing healthcare needs; Fracture related healthcare needs; fall risk assessment; Prevention strategies that need to be put in place prior to discharge; Functional (Activities of Daily Living and Instrumental Activities of Daily Living) needs identified and how they will be met at the time of discharge; Social care needs that need to be in place prior to discharge; Appropriate connection to community resources as required; Appropriate communication to primary care provider and institutions as needed at the time of discharge; and, Plans for needed follow up with Orthopedics and primary care providers. The patient navigator will be available to the patient while the care plan is being enacted and up to the point of three months post-discharge.

At the time of discharge from the OU (defined as any period within 72 hours), all patients will be asked to complete the CPES-IC and a family member responsible for the care of the patient will be asked to complete the CANHELP-L-CQ. The number of nights spent in acute care and the PFFS (current state) will be recorded. The patient and/or family caregiver will be given a form to help them keep track of healthcare utilization after discharge. The patient or family member will be contacted once a month for three months to understand and collect the information about the breakdown of healthcare utilizations that occurred during the previous month. The final HU will be collected at the end of the third month post OU discharge and a total HU will be recorded. If the patient requires readmission during the three-month period and are in the intervention group, the patient navigator will continue to provide navigation services as needed. Should the patient

die over the course of the research project, either as an in-patient or after discharged from acute care, the data collected up until that point would be used within the analysis, as appropriate. At three months post-discharge from acute care, patients and/or family caregivers will be contacted by a research team member to conduct a semi-structured interview about their experiences transitioning from acute care and to complete the global satisfaction with healthcare question. The semi-structured interviews will be audio recorded.

Once recruitment of patients for the study has ended, healthcare providers who worked with the patient navigators in any capacity, such as on the OU and in any receiving facility (other hospital, nursing home, special care home), and the patient navigators themselves, will be approached about participating in semi-structured interviews. HCP will self-refer to participate in the project. Posters will be put in HCP areas of the OU unit and snowball methodology will also be used, asking HCP to forward research team contact information to those other HCP they think may be interested in participating. HCP will be provided with the Healthcare Provider consent form to review (see Appendix B). Healthcare providers who provide informed consent will be asked to participate in a telephone or in-person semi-structured interview with a research team member (other than the patient navigators), which will take approximately 20 to 30 minutes and will be audio recorded. See Table 1 for an outline of the schedule of data collection events for the research study.

Table 1

Schedule of Data Collection Events for the Present Study

	**Time of consent**	**Discharge from OU**	**3 Months Post-Discharge**
**Measures**			
Patient			
Demographics	✓		
CGA	✓*		
PFFS	✓**	✓	✓
CPES-IC		✓	
Global PS			✓
Final HU			✓
Caregiver			
Demographics	✓		
CANHELP-L-CQ		✓	
**Semi-Structured Interviews**			✓

Note. OU = Orthopedic Unit; CGA = Comprehensive Geriatric Assessment; PFFS = Pictorial Fit-Frail Scale; CPES-IC = Canadian Patient Experiences Survey Inpatient Care; Global PS = Global Patient Satisfaction; HU = Healthcare Utilization; CANHELP-L-CQ = CANHELP Lite Family Caregiver Questionnaire.

*The CGA will only be completed for patients in the Patient Navigator group.

**At the time of consent, the patient's current frailty and usual frailty [i.e., frailty prior to present hospitalization] will be recorded.

Data will be kept in password protected files on Horizon Health Network password protected computers or encrypted password protected data keys when being transferred between team members and destroyed after seven years.

Design

The research study uses a concurrent embedded mixed methods design, in which the quantitative randomized control trial has an embedded qualitative component.[45]

A randomized control trial will be conducted in the OU at SJRH. Patients who meet the inclusion criteria will be consented and assigned to either the Patient Navigator group (PN) or standard of care group (SOC) using simple randomization via an online generator (RANDOM.ORG - List Randomizer) and a 1:1 ratio. Blinding will not be possible; thus, increasing the likelihood of post-randomization confounding. For instance, patients assigned to the SOC group may report lower satisfaction and less positive experiences simply because they know they did not receive the care of the PN. However, bias due to factors such as non-compliance will be minimized by using intention-to-treat analysis. All participants will be analyzed in their originally assigned groups regardless of whether they complied with the PN plan or dropped out of the study altogether. Loss-to-follow up will be examined to determine if it is associated with other study variables.

Quantitative Data Analyses

Data will be checked for data entry errors. The procedures outlined in Tabachnick and Fidell[46] will be used to determine if missing data are missing at random (MAR) and to ensure that all assumptions (e.g., normality) are met. If missing data are MAR, missing data will be imputed using multiple imputation in Mplus. Data will also be screened prior to analysis. Tabachnick and Fidell's[46] procedures for screening grouped data will be used. Groups will be checked for balance in baseline characteristics such as frailty, age, and gender.

Study variables and participant demographics, medical, hospital stay, and care plan descriptives will be summarized. Continuous variables will be summarized using means and standard deviations when distributions are normal and medians with interquartile ranges where distributions are not normal. Categorical variables will be summarized using percentages and frequencies. A table of PFFS scores over time (prior to fracture, at time of consent, at time of discharge from OU, at 3 months post-discharge) and by group (PN, SOC) will be created.

Structural Equation Modeling (SEM) will be used to test outcomes following discharge and follow-up. SEM is a comprehensive statistical approach to testing complex relationships. Breitsohl[47] noted advantages of SEM over traditional analyses of experimental design (e.g., ANOVA). Notably, they include an integrated approach to assessing the measurement model,

hypotheses, and measurement error. Further, because measurement error is assessed and removed, power to detect a difference is increased.

Figure 2 illustrates the three outcome variables and three predictor variables in the structural equation model assessing patient outcomes at acute care discharge. Two of these three outcome variables are latent variables and one is an observed variable. The outcome variables are PE, CE and LOS, respectively. The primary predictors are PN (PN vs. SOC) and frailty. A secondary predictor is the moderator effect. If SEM fails to produce an admissible solution, the PE and FE outcomes may be modelled separately from the LOS outcomes, or the two-step estimation and factor score regression with Croon's correction as recommended by Rosseel[48,49] may be used. If sample size is not large enough to obtain a solution without errors, three point-biserial correlations will assess the differences in PE, CE, and LOS.

The second SEM will examine outcomes at follow-up. These outcomes are illustrated in Figure 3 and include PS and HU. Patient Satisfaction will be measured with one question assessing the patient's overall satisfaction with the healthcare they received in the three-month follow-up period. The two predictor variables are frailty and PN. Similar to the first SEM, a secondary predictor is the moderator effect. The analysis will be the same as the first SEM noted above. If sample size is not large enough to obtain a solution without errors, two point-biserial correlations will assess the differences in HU and PS.

Qualitative Data Analysis

The qualitative data from patients/family caregivers and healthcare providers will be iteratively transcribed verbatim, by a research team member or transcription service with appropriate privacy safeguards, and uploaded to NVivo, a qualitative analysis software. The interview transcripts will be deidentified. Audio recordings will be destroyed once transcripts have been verified and deidentified by a research team member. These data for each participant group will then be separately subjected to an iterative thematic analysis[50]. Patient Navigator data will be included with HCP data in order to ensure anonymity. The analysis and the reporting of it will ensure Patient Navigators are not able to be identified. Team analysis will be conducted by qualitatively trained members of the research team, and themes agreed upon through consensus. These results will then be compared between patients that received a patient navigator and those patients that did not, and both will be compared to results from the healthcare provider analysis.

Conclusion

Fall-related injuries like fractures are on the rise among older adults in New Brunswick. These injuries can lead to hospitalization and adverse health effects. Moreover, transitions from acute care can be complicated and overwhelming, especially for patients and families. Researching patient navigators as a means of enhancing inpatient care, while also ensuring successful transitions in care for patients, may have positive impacts and help older adults successfully age in place. This study seeks to investigate the effects of having support from patient navigators in helping older adults admitted to the Orthopedic Unit with a fracture. Specifically, the objectives are to investigate whether there are differences between patients with patient navigators and

those patients receiving standard of care, regarding: the length of stay in acute care; healthcare utilization post-discharge; patient and family experience and satisfaction with care; and, healthcare provider experiences working with patient navigators. This study uses a mixed-method concurrent embedded mixed methods design, in which the quantitative randomized control trial has an embedded qualitative component. Potential benefits include a better understanding of the impact of support from a patient navigator on inpatient care and patient transitions. This information will be used to inform the development of practical recommendations for policymakers and clinicians on how to enhance inpatient care and successful transitions for older adults.

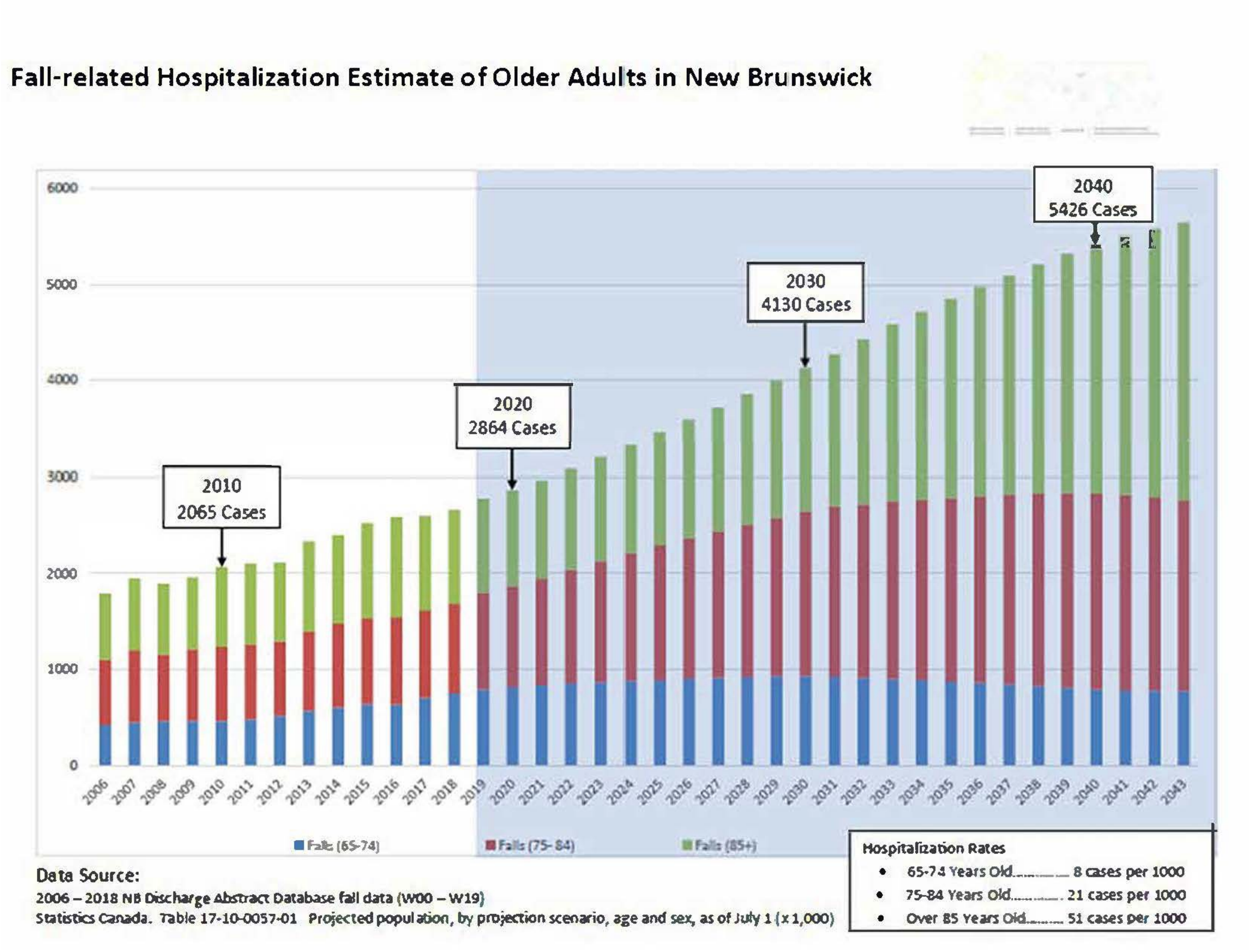

Figure 1. Fall-related hospitalization estimate of older adults in New Brunswick.

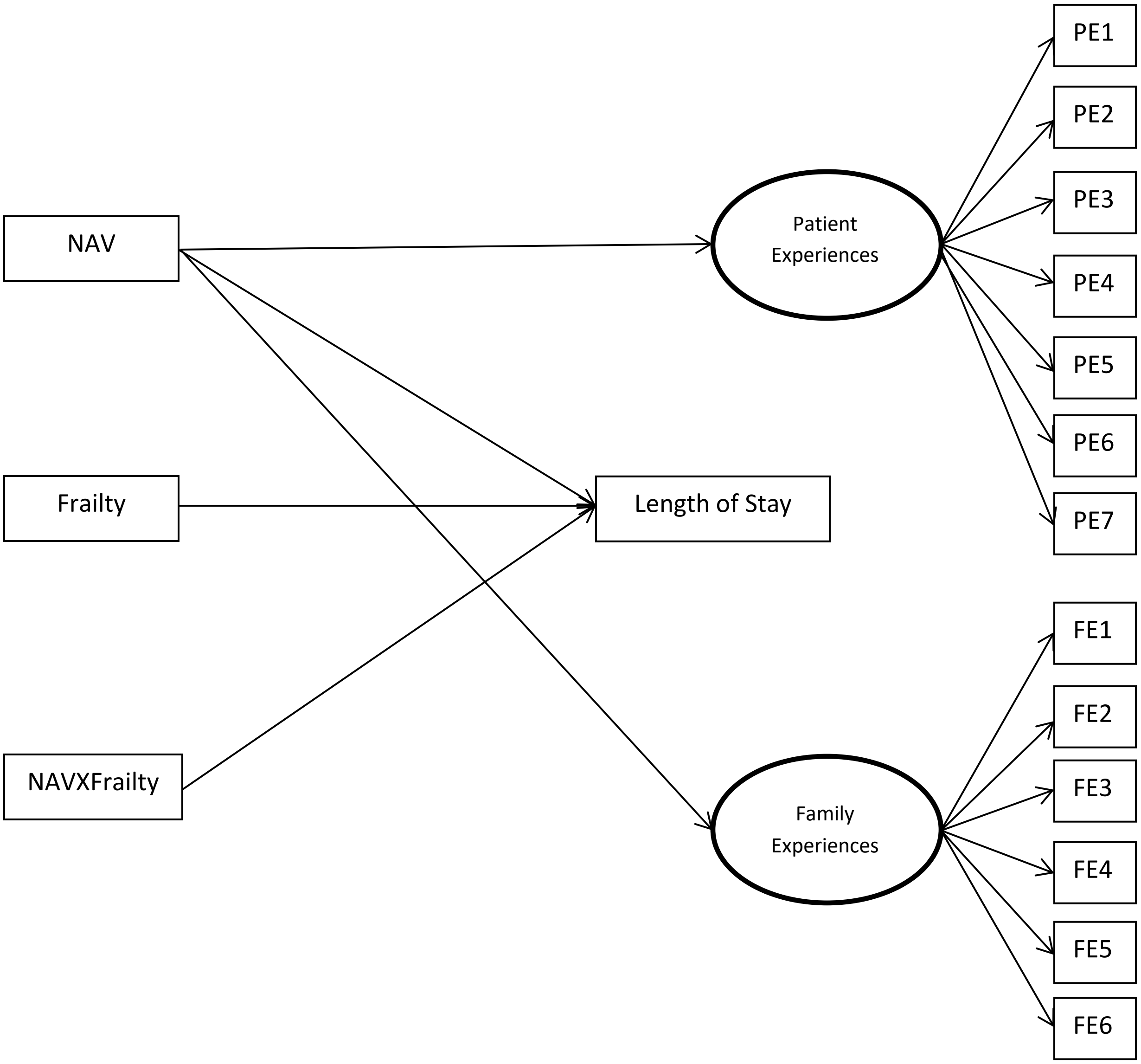

Figure 2. Patient navigator vs. standard of care and frailty predicting patient experiences, family experiences and length of stay.

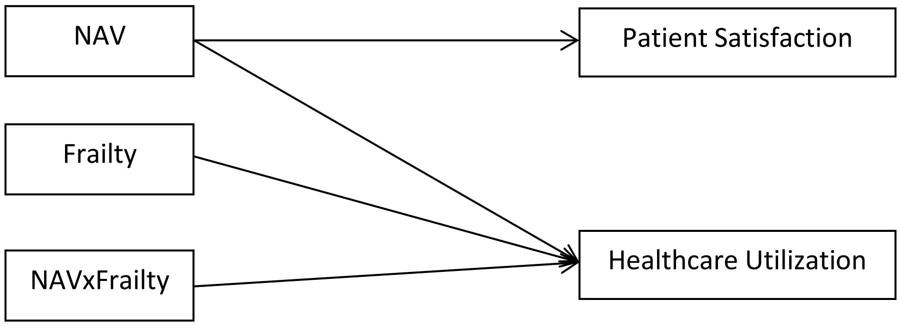

Figure 3. Patient navigator vs. standard of care and frailty predicting patient satisfaction and healthcare utilization.

Appendix A: Codebook and Measures

**Label**	**Abbreviation**	**Operationalization/Code**
Patient: ID Numbers		
Identifying Number	id	To ID case without HCN
Health Care Number	hcn	To connect records
Random Group Assignment	rga	0=Standard of Care<br>1=Patient Navigator
**Patient: Demographics**		
Age at time of admission	age_p	
Gender	gen_p	0=Male<br>1=Female<br>2=Other
Ethnicity	eth_p	1= First Nation<br>2= Inuit<br>3= Métis<br>4= Indigenous/Aboriginal (not included above)<br>5= Arab<br>6= Black (North American, Caribbean, African, etc.)<br>7= Chinese<br>8= Filipino<br>9= Japanese<br>10= Korean<br>11= Latin American<br>13= South Asian (East Indian, Pakistani, Sri Lankan, etc.)<br>14= Southeast Asian (Vietnamese, Cambodian, Malaysian, Laotian, etc.)<br>15= West Asian (Iranian, Afghan, etc.)<br>16= White (North American, European, etc.)<br>17= Other
Education Level	edu_p	1= 8th grade or less<br>2=Some high school, but did not graduate<br>3=High school or high school equivalency certificate

		4= College, CEGEP or other non-university certificate or diploma<br>5= Undergraduate degree or some university<br>6= Post-graduate degree or professional designation
Marital Status	mar_p	1=married<br>2=common-law partner<br>3=divorced/separated<br>4=widowed<br>5=single
Patient: Frailty		
PFFS: Frailty Prior to Fracture	PFFS1	##
PFFS: Frailty At Time of Consent	PFFS2	##
PFFS: Frailty at Time of Discharge from OU	PFFS3	##
PFFS: Frailty at Time of Follow-up	PFFS4	##
Patient: Medical and Hospital Stay Information		
Date of Hospital Admission	dha	dd/mm/yyyy
Date of Discharge_Acute Care	ddac	dd/mm/yyyy
Date of Discharge from Hospital	ddh	dd/mm/yyyy
Reason for Hospital Admission	rha	Open text
Treatment During Hospital Stay*	treat	Open text
Primary Diagnosis	pd	Open text
Secondary Diagnoses*	sd	Open text
Pre-Admit Comorbidities*	pac	Open text
Medications*	med	Open text
Post-Discharge AU Location(s)*	pdl	Open text
Length of Time at that Location*	ltl	#-### nights
Consultations*	cons	Open text
Other*	oth	Open text
**Patient: Canadian Patient Experiences Scale**		
Communication with Doctors:<br>*During this hospital stay, how often did doctors treat you with courtesy and respect?*	Q5	0=Never<br>1=Sometimes<br>2=Usually<br>3=Always
Communication with Doctors:<br>*During this hospital stay, how often did doctors listen carefully to you?*	Q6	0=Never<br>1=Sometimes<br>2=Usually<br>3=Always
Communication with Doctors:	Q7	0=Never<br>1=Sometimes

During this hospital stay, how often did doctors explain things in a way you could understand?		2=Usually<br>3=Always
Communication with Nurses:<br>*During this hospital stay, how often did nurses treat you with courtesy and respect?*	Q1	0=Never<br>1=Sometimes<br>2=Usually<br>3=Always
Communication with Nurses:<br>*During this hospital stay, how often did nurses listen carefully to you?*	Q2	0=Never<br>1=Sometimes<br>2=Usually<br>3=Always
Communication with Nurses:<br>*During this hospital stay, how often did nurses explain things in a way you could understand?*	Q3	0=Never<br>1=Sometimes<br>2=Usually<br>3=Always
Discharge Management:<br>*Before you left the hospital, did you have a clear understanding about all of your prescribed medications, including those you were taking before your hospital stay?*	Q37	0=Not at all<br>1=Partly<br>2=Quite a bit<br>3=Completely
Discharge Management:<br>*Did you receive enough information from hospital staff about what to do if you were worried about your condition or treatment after you left the hospital?*	Q38	0=Not at all<br>1=Partly<br>2=Quite a bit<br>3=Completely
Discharge Management:<br>*When you left the hospital, did you have a better understanding of your condition than when you entered?*	Q39	0=Not at all<br>1=Partly<br>2=Quite a bit<br>3=Completely
Emotional Support:<br>*Did you get the support you needed to help you with any anxieties, fears or worries you had during this hospital stay?*	Q34	0=Never<br>1=Sometimes<br>2=Usually<br>3=Always
Internal Coordination of Care:<br>*Do you feel that there was good communication about your care between doctors, nurses and other hospital staff?*	Q30	0=Never<br>1=Sometimes<br>2=Usually<br>3=Always
Internal Coordination of Care:<br>*How often did doctors, nurses and other hospital staff seem informed and up-to-date about your hospital care?*	Q31	0=Never<br>1=Sometimes<br>2=Usually<br>3=Always
Involvement in Decision Making:<br>*Were you involved as much as you wanted to be in decisions about your care and treatment?*	Q35	0=Never<br>1=Sometimes<br>2=Usually<br>3=Always

Involvement in Decision Making:<br>*Were your family or friends involved as much as you wanted in decisions about your care and treatment?*	Q36	0=Never<br>1=Sometimes<br>2=Usually<br>3=Always<br>4=I did not want them to be involved<br>5=I did not have family or friends to be involved
Overall Hospital Experience:<br>*Overall , I had a ? experience.*	Q41	0=Very Poor<br>1-9<br>10=Very Good
Patient: Other Outcomes		
Length of Stay (# of nights in acute care)	LOS	#-##
Healthcare Utilization:<br>Number of unplanned healthcare utilizations during 3mth follow-up	HU	#-##
Patient Satisfaction:<br>*"Thinking about all of your healthcare experiences related to your fracture of the last 3 months, to what degree are you satisfied or dissatisfied with the services you have received?"*	PS	0=Not at all Satisfied<br>1-9<br>10=Very Satisfied
**Family Caregiver: Demographics**		
Age XXXX	Age	Age ###
Gender	gen_c	0=Male<br>1=Female<br>2=Other
Ethnicity	eth_c	1= First Nation<br>2= Inuit<br>3= Métis<br>4= Indigenous/Aboriginal (not included above)<br>5= Arab<br>6= Black (North American, Caribbean, African, etc.)<br>7= Chinese<br>8= Filipino<br>9= Japanese

		10= Korean<br>11= Latin American<br>13= South Asian (East Indian, Pakistani, Sri Lankan, etc.)<br>14= Southeast Asian (Vietnamese, Cambodian, Malaysian, Laotian, etc.)<br>15= West Asian (Iranian, Afghan, etc.)<br>16= White (North American, European, etc.)<br>17= Other
Education Level	edu_c	1= 8th grade or less<br>2=Some high school, but did not graduate<br>3=High school or high school equivalency certificate<br>4= College, CEGEP or other non-university certificate or diploma<br>5= Undergraduate degree or some university<br>6= Post-graduate degree or professional designation
Marital Status	mar_c	1=married<br>2=common-law partner<br>3=divorced/separated<br>4=widowed<br>5=single
Relationship to patient	rel	**Operationalization/Code**<br>1=Spouse/Partner<br>2=Parent<br>3=Adult Child<br>4=Friend<br>5=Family Member (sister/brother/uncle/aunt)<br>6=Other. Please, specify:
**Family Caregiver: CANHELP-L-CQ**		
Overall Satisfaction: *In general, how satisfied are you with the*	CQ1	1=Not at all Satisfied<br>2=Not Very Satisfied

quality of care your relative received?		3=Somewhat Satisfied<br>4=Very Satisfied<br>5=Completely Satisfied
Overall Satisfaction*: In general, how satisfied are you with the way you were treated by the doctors, nurses, and other healthcare professionals looking after your relative?*	CQ2	1=Not at all Satisfied<br>2=Not Very Satisfied<br>3=Somewhat Satisfied<br>4=Very Satisfied<br>5=Completely Satisfied
Relationship with the Doctors: *How satisfied are you that the doctor(s) took a personal interest in your relative?*	CQ3	1=Not at all Satisfied<br>2=Not Very Satisfied<br>3=Somewhat Satisfied<br>4=Very Satisfied<br>5=Completely Satisfied
Relationship with the Doctors: *How satisfied are you that the doctor(s) were available when you or your relative needed them (by phone or in person).*	CQ4	1=Not at all Satisfied<br>2=Not Very Satisfied<br>3=Somewhat Satisfied<br>4=Very Satisfied<br>5=Completely Satisfied
Relationship with the Doctors: *How satisfied are you with the level of trust and confidence in the doctor(s) who looked after your relative.*	CQ5	1=Not at all Satisfied<br>2=Not Very Satisfied<br>3=Somewhat Satisfied<br>4=Very Satisfied<br>5=Completely Satisfied
Characteristics of the Doctors and Nurses: *How satisfied are you that the doctors, nurses, and other healthcare professionals looking after your relative were compassionate and supportive of him or her.*	CQ6	1=Not at all Satisfied<br>2=Not Very Satisfied<br>3=Somewhat Satisfied<br>4=Very Satisfied<br>5=Completely Satisfied
Characteristics of the Doctors and Nurses: *How satisfied are you that the doctors, nurses, and other healthcare professionals looking after your relative were compassionate and supportive of you.*	CQ7	1=Not at all Satisfied<br>2=Not Very Satisfied<br>3=Somewhat Satisfied<br>4=Very Satisfied<br>5=Completely Satisfied
Illness Management: *How satisfied are you with the tests that were done and the treatments that were given to treat your relative's medical problems.*	CQ8	1=Not at all Satisfied<br>2=Not Very Satisfied<br>3=Somewhat Satisfied<br>4=Very Satisfied<br>5=Completely Satisfied

Illness Management: *How satisfied are you that the physical symptoms (for example, pain, shortness of breath, nausea) your relative had were adequately controlled.*	CQ9	1=Not at all Satisfied<br>2=Not Very Satisfied<br>3=Somewhat Satisfied<br>4=Very Satisfied<br>5=Completely Satisfied
Illness Management: *How satisfied are you that the emotional problems (for example: depression, anxiety) your relative had were adequately controlled.*	CQ10	1=Not at all Satisfied<br>2=Not Very Satisfied<br>3=Somewhat Satisfied<br>4=Very Satisfied<br>5=Completely Satisfied
Illness Management: *How satisfied are you with the help your relative received for personal care (for example: bathing, toileting, dressing, eating) when needed.*	CQ11	1=Not at all Satisfied<br>2=Not Very Satisfied<br>3=Somewhat Satisfied<br>4=Very Satisfied<br>5=Completely Satisfied
Illness Management: *How satisfied are you that your relative received good care when you were not able to be with him/her.*	CQ12	1=Not at all Satisfied<br>2=Not Very Satisfied<br>3=Somewhat Satisfied<br>4=Very Satisfied<br>5=Completely Satisfied
Illness Management*: How satisfied are you that the healthcare workers worked together as a team to look after your relative.*	CQ13	1=Not at all Satisfied<br>2=Not Very Satisfied<br>3=Somewhat Satisfied<br>4=Very Satisfied<br>5=Completely Satisfied
Illness Management: *How satisfied are you that you were able to manage the financial costs associated with your relative's illness.*	CQ14	1=Not at all Satisfied<br>2=Not Very Satisfied<br>3=Somewhat Satisfied<br>4=Very Satisfied<br>5=Completely Satisfied
Illness Management: *How satisfied are you that the environment or the surroundings in which your relative was cared for was calm and restful.*	CQ15	1=Not at all Satisfied<br>2=Not Very Satisfied<br>3=Somewhat Satisfied<br>4=Very Satisfied<br>5=Completely Satisfied
Illness Management*: How satisfied are you that the care and treatment your relative received was consistent with his or her wishes.*	CQ16	1=Not at all Satisfied<br>2=Not Very Satisfied<br>3=Somewhat Satisfied<br>4=Very Satisfied<br>5=Completely Satisfied
Communication & Decision Making: *How satisfied are you that the doctor(s) explained things relating to your relative's illness in a straightforward, honest manner.*	CQ17	1=Not at all Satisfied<br>2=Not Very Satisfied<br>3=Somewhat Satisfied<br>4=Very Satisfied<br>5=Completely Satisfied

Communication & Decision Making: *How satisfied are you that you received consistent information about your relative's condition from all the doctors and nurses looking after him or her.*	CQ18	1=Not at all Satisfied<br>2=Not Very Satisfied<br>3=Somewhat Satisfied<br>4=Very Satisfied<br>5=Completely Satisfied
Communication & Decision Making: *How satisfied are you that the doctor(s) listened to what you had to say.*	CQ19	1=Not at all Satisfied<br>2=Not Very Satisfied<br>3=Somewhat Satisfied<br>4=Very Satisfied<br>5=Completely Satisfied
Communication & Decision Making: *How satisfied are you with discussions with the doctor(s) about where your relative would be cared for (in hospital, at home, or elsewhere) if he or she were to get worse.*	CQ20	1=Not at all Satisfied<br>2=Not Very Satisfied<br>3=Somewhat Satisfied<br>4=Very Satisfied<br>5=Completely Satisfied
Your Involvement: *How satisfied are you with discussions with the doctor(s) about the use of life sustaining technologies (for example: CPR or cardiopulmonary resuscitation, breathing machines dialysis).*	CQ21	1=Not at all Satisfied<br>2=Not Very Satisfied<br>3=Somewhat Satisfied<br>4=Very Satisfied<br>5=Completely Satisfied
Your Involvement: *How satisfied are you with your role in decision-making regarding your relative's medical care.*	CQ22	1=Not at all Satisfied<br>2=Not Very Satisfied<br>3=Somewhat Satisfied<br>4=Very Satisfied<br>5=Completely Satisfied
Your Involvement: *How satisfied are you with discussions with your relative about his/her wishes for future care in the event he or she is unable to make those decisions.*	CQ23	1=Not at all Satisfied<br>2=Not Very Satisfied<br>3=Somewhat Satisfied<br>4=Very Satisfied<br>5=Completely Satisfied

- These items may require more than one column. If so, numbers will be added to their code name (e.g., sd1, sd2, sd3).

CHARM Study

Patient Demographic Questionnaire

Patient Contact Information

Name (full): ____

Home phone number: ____

Cell phone number: ____

Email address: ____

Healthcare number: ____

Patient Demographic Information

Gender
(Circle one)

Female
Male
Other (please specify) __________________

Age at time of admission __________________ (years)

Race/Ethnicity:
(Circle one)

First Nation
Inuit
Métis
Indigenous/Aboriginal (not included above)
Arab
Black (North American, Caribbean, African, etc.)
Chinese
Filipino
Japanese
Korean
Latin American
South Asian (East Indian, Pakistani, Sri Lankan, etc.)
Southeast Asian (Vietnamese, Cambodian, Malaysian, Laotian, etc.)
West Asian (Iranian, Afghan, etc.)
White (North American, European, etc.)
Other (please specify) __________________

**Marital status (Circle one)**	Married<br>Common-law partner<br>Divorced/separated<br>Widowed<br>Single
**Education level (Circle one)**	8th grade or less<br>Some high school, but did not graduate<br>High school or high school equivalency certificate<br>College, CEGEP or other non-university certificate or diploma<br>Undergraduate degree or some university<br>Post-graduate degree or professional designation

CHARM Study

Family Caregiver Demographic Questionnaire

Caregiver Contact Information

Name (full): ____

Home phone number: ____

Cell phone number: ____

Email address: ____

Caregiver Demographic Information

Gender
(Circle one)

Female
Male
Other (please specify) __________________

Age ____________(years)

Race/Ethnicity:
(Circle one)

First Nation
Inuit
Métis
Indigenous/Aboriginal (not included above)
Arab
Black (North American, Caribbean, African, etc.)
Chinese
Filipino
Japanese
Korean
Latin American
South Asian (East Indian, Pakistani, Sri Lankan, etc.)
Southeast Asian (Vietnamese, Cambodian, Malaysian, Laotian, etc.)
West Asian (Iranian, Afghan, etc.)
White (North American, European, etc.)
Other (please specify) __________________

Marital status **(Circle one)**

Married
Common-law partner
Divorced/separated
Widowed
Single

Education level **(Circle one)**

8th grade or less
Some high school, but did not graduate
High school or high school equivalency certificate
College, CEGEP or other non-university certificate or diploma
Undergraduate degree or some university
Post-graduate degree or professional designation

What is your relationship with the patient?

- ☐ Spouse/Partner
- ☐ Parent
- ☐ Adult child
- ☐ Friend
- ☐ Family Member (sister/brother/uncle/aunt)
- ☐ Other (please specify) ________________________

Patient Health Care Utilization

Instructions: This form is to be completed by either the study participant or their family caregiver. All information should be kept track of as accurately as possible and completed immediately following each visit.

This questionnaire was completed by the ________ patient _________ family caregiver

Visit to Emergency Department	Date	Time	Reason for visit	Duration of visit
Readmission to Hospital	Date	Time	Reason for visit	Duration of visit
Visit to Family Doctor	Date	Time	Reason for visit	Duration of visit
Call to Paramedics	Date	Time	Reason for visit	Duration of visit
Other (*Examples*: Telecare:811; After-hours clinic visit)	Date	Time	Reason for visit	Duration of visit

Comprehensive Geriatric Assessment

PATIENT LABEL

Date: ____________________

CMH: ______ DSD: ______ DVA: ______

Code Status: ____________ Case Worker: ____________________

History of Illness:

Patient Status:
- ☐ Inpatient
- ☐ Clinic
- ☐ Home
- ☐ NH
- ☐ Other

Comprehensive Geriatric Assessment

**Mental Status**		WNL CIND/MCI Dementia Delirium MMSE: ____ MoCA: ____ FAST: ____ Education (yrs): ____
**Emotional**		WNL ↓Mood Depression Anxiety Poor Motivation Hallucinations Delusions Other<br>GDS:
**Communication**		Speech: WNL Impaired Hearing: WNL Impaired Vision: WNL Impaired
**Mobility**	PTA	Transfers: I A D Walking: I A D Aids: ____________ ☐ Level home ☐ ______ Steps
	Current	Transfers: I A D Walking: I A D Aids: ____________
**Balance**		WNL Impaired Fall(s): ☐Yes ☐ No Frequency: ____________
**Elimination**		Bowel: Continent Incontinent Constipation<br>Bladder: Continent Incontinent Catheter
**Nutrition**		Appetite: WNL Fair Poor Weight (kg): ______ Stable Loss Gain (______ Kg)
**Function**	PTA	Bathing: I A D Dressing: I A D Toileting: I A D<br>Cleaning: I A D Cooking I A D Shopping: I A D<br>Medications: I A D Banking: I A D Telephone: I A D<br>Driving: ☐Yes ☐ No
	Current	Bathing: I A D<br>Dressing: I A D<br>Toileting: I A D
**Sleep**		Normal Disrupted Daytime drowsiness
**Social**		☐ Married ☐ Single ☐ Widowed ☐ Divorced ☐ Children #: ____<br>Lives: ☐ Alone ☐ Spouse ☐ Other<br>Supports: ☐ None ☐ Informal ☐ Formal ___ hrs/day ___ days/wk ☐ Requests more<br>Home: ☐ House ☐ Apt ☐ SCH ☐ Nursing home ☐ Assisted Living<br>Primary Caregiver: ☐ Spouse ☐ Sibling ☐ Offspring ☐ Other<br>Caregiver Stress: ☐ None ☐ Low ☐ Moderate ☐ High<br>ETOH: ☐Yes ☐ No Smoking: ☐Yes ☐ No POA: ☐Yes ☐ No<br>Financial POA: ____________<br>Personal POA: ____________

Clinical Global Frailty Score: ☐ Very fit ☐ Well ☐ Well, with treated co-morbid disease ☐ Apparently vulnerable ☐ Mildly frail ☐ Moderately frail ☐ Severely frail

33680 NS (05/09)

**Medical and Surgical Problems**	**Associated Medications** (at home)	(in hospital)

Family History: ______________________________

Known Drug Allergies:

Physical Examination

Appearance:

Vital Signs:

Head & Neck:

Respiratory:

Cardiovascular:

Abdomen:

Joints/Extremities:

Skin:

CNS:

Cranial Nerves

Motor/Tone/Sensation

Cerebellum

Gait

Reflexes

Mini Mental Status Exam (MMSE) **Total:** ____ / **30** **Date:** ____________

1. Orientation

Vhat is today's date (year) (season) (date) (day) (month)?... (5 pts) ____

Where are we (country) (province) (city) (hospital) (floor)?... (5 pts) ____

Draw a clock

2. Registration

Name 3 objects (apple, table, penny)........................ (3 pts) ____

3. Attention

Spell world backwards .... (5 pts) ____

4. Recall

Repeat the three objects above .... (3 pts) ____

5. Language

Name: pencil, watch .... (2 pts) ____

Repeat: "No ifs, ands, or butts" .... (1 pt) ____

Follow a three-stage command: .... (3 pts)

"Take the paper in your right/left hand, fold it in half and put it on the floor" ____

Read and obey .... (1 pt) ____

CLOSE YOUR EYES

Write a sentence: ________________________________ (1 pt) ....____

PLEASE WRITE A SENTENCE BELOW

6. Visuospatial

Copy pentagon: .... (1 pt) ....____

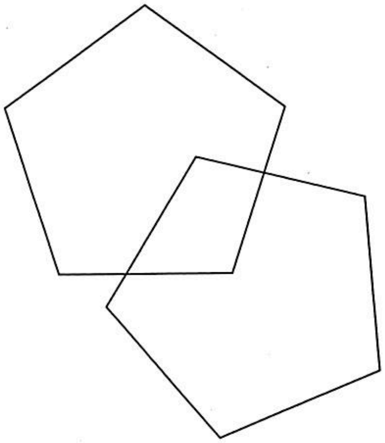

Assessor/Physician: ______________________________

Problem List

Assessor/Physician: ______________________ **Date:** ______________
yyyy/mm/dd

Appendix B

Participant Consent Forms

STUDY SPECIFIC INFORMED CONSENT FORM
Patients

TITLE OF STUDY/PROTOCOL: The CHARM Study - Coordinating transitions from hospital for older adults with fractures: An interventional mixed methods study.

PRINCIPAL INVESTIGATOR: Ian Watson, BSc, MHSc
Administrative Director, Trauma NB
Horizon Health Network, Saint John, NB

FILE NUMBER:

PURPOSE

This study is testing the effects of having support from patient navigators in helping older adults admitted to the Orthopedic Unit with a fracture. The study will look to see if there are differences between patients who are assigned patient navigators versus patients receiving usual care (no patient navigator assigned) when it comes to healthcare usage, experiences and satisfaction. The goal of this study is to enhance inpatient care and ensure successful transitions in care for patients and families.

STUDY DESIGN

This study will start June 20, 2022 and finish March 31, 2023. You are being asked to be a part of this study because you are a patient 65 years of age or older who was admitted to the Orthopedic Unit at the Saint John Regional Hospital with a fracture. We are looking to recruit a total of 60 patients for this study. Patients will be randomly assigned to either the Patient Navigator group or standard of care group. For patients in the patient navigator group, the navigator will work with the patient's healthcare team to develop a patient specific and attainable care plan needed for a successful discharge from acute care. Family caregivers and healthcare providers will also be recruited.

Participation in this study means that the research team will follow you through your care in the Orthopedic Unit, through your transition out of acute care, and for three months after leaving the unit. Data will be collected by interviewing you, asking questions from surveys, and through your medical records. A notification letter of your enrolment in the study will be sent to your primary healthcare provider to make them aware of your participation.

The following general information will be collected about you: Name, contact phone number(s), email address, health care number, gender, age at time of admission, marital status, race/ethnicity, and education. Also, the following information will be collected from your medical record concerning your latest hospital admission: date of hospital admission; reason for hospital admission; treatment; diagnoses; medications; discharge dates (from acute care unit and from hospital); locations post-discharge and length; and, documentation associated with care transitions (including patient navigator care plan), also including consultations placed to community agencies by hospital staff and discharge instructions.

You are being asked to have questionnaires collected from you by a research assistant and/or the Patient Navigator, depending on which group you are randomly assigned to. These measures include assessments of clinical frailty, patient experiences in acute care settings, and global patient satisfaction. Patients in the Patient Navigator group will also have a Comprehensive Geriatric Assessment conducted on them by the Patient Navigator. The Comprehensive Geriatric Assessment collects information on medical, functional, psychological, and social assessments from the patient and/or family members and health care team members, when relevant.

You are being asked to track your healthcare use on an ongoing basis for three months after you are discharged from acute care. Examples of healthcare visits that will be tracked include visits to the Emergency Department, Family Doctor, or the After-Hours Clinic. Both you and your identified family caregiver will be given a Health Care Utilization form and instructions for keeping track of all visits or consultations during the three-month period. You or your family caregiver will be contacted once a month for three months to understand and collect the information about the breakdown of healthcare visits that occurred during the previous month.

Someone from the research team will interview you at a time and place that works for you or over the telephone if you prefer. The interview will take place around three months after your discharge from acute care. The interview will take about 30 to 60 minutes. You can talk longer or shorter if you want. It is up to you to decide whether you would like to do the interview either separately, or together with the person you have identified as your caregiver. Patients in the Patient Navigator group will be asked about their experiences with the Patient Navigator. All patients will be asked about their experiences in transitioning from acute care and about any improvements that could be made. The information that you share will be audio recorded, then typed up and kept private. Anything that could identify you will be taken out.

If you want, the results of the study will be shared with you. This can help make sure that the findings are correct. If you would like to find out the results of the study, please check the box below:

- □ Yes, I would like to be contacted with the study results.

Contact information: ____

If you do not wish to know the results, please check the box below:

- □ No, please do not contact me.

VOLUNTARY PARTICIPATION

Being part of this study is completely up to you. You can stop at any time, for any reason. There will be no negative changes to your medical care if you decide not to take part in this study. If you decide to stop being part of the study, you will be asked why but it is up to you whether you tell them your reasons. You may also tell the research team what information that you want them to use and not use in the study, up to the point where the data is analysed. After the analysis your information cannot be removed.

PRIVACY AND CONFIDENTIALITY

Privacy: All your information will be kept private and safe. All interview materials will have information that could identify you removed from them during transcription. Only the research team members will be able to read all the information collected. The research team will follow all privacy and confidentiality laws and policies.

Publication: The results of the study may be made public. You will not be identified. Your private information will not be shared with others without your permission. Your name will not appear in any writing made public.

Information Retention: Information collected for the research study will be kept for 7 years once the research is done and the results made public. Your information will be kept secure by the research team at the site where the study is being conducted.

RISKS AND POTENTIAL BENEFITS

This study is considered low risk. You may become upset during the interview. If you become upset, you can stop the interview. The research team member will ask you if you need to follow up with someone to help you. Positive things that could come from this research include helping to understand the impact of support from a patient navigator on inpatient care and patient transitions. This information will be used to inform the development of practical recommendations for policy makers and clinicians on how to enhance inpatient care and successful transitions for older adults.

RESEARCH RELATED INJURY

If you become sick or hurt by taking part in this study, you will receive medical care at no cost to you. By signing this form, you are not waiving your legal rights or freeing the Principal Investigator from their legal responsibilities.

CONTACT INFORMATION

If you have any questions about your rights as someone who takes part in a research study, you may contact the Horizon Health Network Regional Director of Ethics Services at (506)648-6094 or by e-mail at REBOffice@HorizonNB.ca.

If you have any questions or concerns about your privacy rights, you may contact the Privacy Officer for Horizon Health Network at the toll-free number 1-877-422-8717.

Research Study Contact Name:

If you have any questions or concerns about what you just read, please contact the Research Coordinator below:

Leanne Skerry
Research Services, Horizon Health Network
Hilyard Place
560 Main St
Building A, 2nd floor
Suite A-200
E2K 1J5
Phone: 506-643-1321
Email: SOAR@HorizonNB.ca

- ☐ A copy of the signed informed consent form has been provided to the participant.

CONSENT TO PARTICIPATE IN RESEARCH STUDY for *The CHARM Study - Coordinating transitions from hospital for older adults with fractures: An interventional mixed methods study.*

PARTICIPANT'S QUESTIONS:

Have you had an opportunity to ask questions and discuss this study?	☐ Yes	☐ No
Are you comfortable with the information that has been provided?	☐ Yes	☐ No
Do you understand that you are free to withdraw from this study?	☐ Yes	☐ No
Do you understand that you will receive a signed copy of this consent?	☐ Yes	☐ No

PARTICIPANT'S STATEMENT

I have read the above information and understand the purpose of the research. I have had the opportunity to ask questions, and all my questions have been answered. By signing, I am indicating that I have reviewed all 5 pages of this document. I hereby give my informed consent to be a participant in this study.

______________________________	______________________________	____________
Printed Name of Participant/ Substitute Decision Maker	Signature of Participant/ Substitute Decision Maker	Date

______________________________	______________________________	____________
Printed Name of Person Conducting Informed Consent Discussion	Signature of Person Conducting Informed Consent Discussion	Date

Family Caregiver

STUDY SPECIFIC INFORMED CONSENT FORM
Caregivers

TITLE OF STUDY/PROTOCOL: The CHARM Study - Coordinating transitions from hospital for older adults with fractures: An interventional mixed methods study.

PRINCIPAL INVESTIGATOR: Ian Watson, BSc, MHSc
Administrative Director, Trauma NB
Horizon Health Network, Saint John, NB

FILE NUMBER:

PURPOSE

This study is testing the effects of having support from patient navigators in helping older adults admitted to the Orthopedic Unit with a fracture. The study will look to see if there are differences between patients who are assigned patient navigators versus patients receiving usual care (no patient navigator assigned) when it comes to healthcare usage, experiences and satisfaction. The goal of this study is to enhance inpatient care and ensure successful transitions in care for patients and families.

STUDY DESIGN

This study will start June 20, 2022 and finish March 31, 2023. You are being asked to be a part of this study because you have been identified as a family caregiver by an older adult aged 65 or older (referred to as the patient in this consent form) who was admitted to the Orthopedic Unit at the Saint John Regional Hospital with a fracture. Patients and healthcare providers will also be recruited. Patients will be randomly assigned to either the Patient Navigator group or standard of care group. For patients in the patient navigator group, the navigator will work with the patient's healthcare team to develop a patient specific and attainable care plan needed for a successful discharge from acute care.

Participation in this study means that the research team will follow you through the patient's care in the Orthopedic Unit, through the transition out of acute care, and for three months once out of the unit. Data will be collected by interviewing you and asking questions from surveys.

The following general information will be collected from you: Name, contact phone number(s), email address, age, gender, ethnicity, education, marital status and relationship to the patient.

You will be asked to complete a questionnaire to assess your experience with the care that your relative received while in hospital, around the time that they leave the Orthopedic Unit.

You are also being asked to track the healthcare use of the patient on an ongoing basis for three months after they are discharged from acute care. Examples of healthcare visits that will be tracked include visits to the Emergency Department, Family Doctor, or the After-Hours Clinic. You and the patient will be given a Health Care Utilization form and instructions for keeping track of all visits or consultations during the three-month period. You or the patient will be contacted once a month for three months to understand and collect the information about the breakdown of healthcare visits that occurred during the previous month.

Someone from the research team will interview you at a time and place that works for you or over the telephone if you prefer. The interview will take place around three months after the patient's discharge from acute care. The interview will take about 30 to 60 minutes. You can talk longer or shorter if you want. You will be asked questions about your experiences in the patient's transition from acute care and about any improvements that could be made. If the patient was assigned a patient navigator, you will also be asked about your experiences with the patient navigator. The information that you share will be audio recorded, then typed up and kept private. Anything that could identify you will be taken out.

If you want, the results of the study will be shared with you. This can help make sure that the findings are correct. If you would like to find out the results of the study, please check the box below:

- ☐ Yes, I would like to be contacted with the study results.

Contact information: ____

If you do not wish to know the results, please check the box below:

- ☐ No, please do not contact me.

VOLUNTARY PARTICIPATION

Being part of this study is completely up to you. You can stop at any time, for any reason. There will be no negative changes for you or your family member's medical care if you decide not to take part in this study. If you decide to stop being part of the study, you will be asked why but it is up to you whether you share your reasons. You may also tell the research team what information that you want them to use and not use in the study, up to the point where the data is analysed. After the analysis your information cannot be removed.

PRIVACY AND CONFIDENTIALITY

Privacy: All your information will be kept private and safe. All interview materials will have information that could identify you removed from them during transcription. Only the research team members will be able to read all the information collected. The research team will follow all privacy and confidentiality laws and policies.

Publication: The results of the study may be made public. You will not be identified. Your private information will not be shared with others without your permission. Your name will not appear in any writing made public.

Information Retention: Information collected for the research study will be kept for 7 years once the research is done and the results made public. Your information will be kept secure by the research team at the site where the study is being conducted.

RISKS AND POTENTIAL BENEFITS

This study is considered low risk. You may become upset during the interview. If you become upset, you can stop the interview. The research team member will ask you if you need to follow up with someone to help you. Positive things that could come from this research include helping to understand the impact of support from a patient navigator on inpatient care and patient transitions. This information will be used to inform the development of practical recommendations for policy makers and clinicians on how to enhance inpatient care and successful transitions for older adults.

RESEARCH RELATED INJURY

If you become sick or hurt by taking part in this study, you will receive medical care at no cost to you. By signing this form, you are not waiving your legal rights or freeing the Principal Investigator from their legal responsibilities.

CONTACT INFORMATION

If you have any questions about your rights as someone who takes part in a research study, you may contact the Horizon Health Network Regional Director of Ethics Services at (506)648-6094 or by e-mail at REBOffice@HorizonNB.ca.

If you have any questions or concerns about your privacy rights, you may contact the Privacy Officer for Horizon Health Network at the toll-free number 1-877-422-8717.

Research Study Contact Name:

If you have any questions or concerns about what you just read, please contact the Research Coordinator below:

Leanne Skerry
Research Services, Horizon Health Network
Hilyard Place
560 Main St
Building A, 2nd floor
Suite A-200
E2K 1J5
Phone: 506-643-1321
Email: SOAR@HorizonNB.ca

☐ A copy of the signed informed consent form has been provided to the participant.

CONSENT TO PARTICIPATE IN RESEARCH STUDY for *The CHARM Study - Coordinating transitions from hospital for older adults with fractures: An interventional mixed methods study.*

PARTICIPANT'S QUESTIONS:

Have you had an opportunity to ask questions and discuss this study?	☐ Yes	☐ No
Are you comfortable with the information that has been provided?	☐ Yes	☐ No
Do you understand that you are free to withdraw from this study?	☐ Yes	☐ No
Do you understand that you will receive a signed copy of this consent?	☐ Yes	☐ No

PARTICIPANT'S STATEMENT

I have read the above information and understand the purpose of the research. I have had the opportunity to ask questions, and all my questions have been answered. By signing, I am indicating that I have reviewed all 5 pages of this document. I hereby give my informed consent to be a participant in this study.

Printed Name of Participant	Signature of Participant	Date

Printed Name of Person Conducting Informed Consent Discussion	Signature of Person Conducting Informed Consent Discussion	Date

Healthcare Provider-Email Consent Form

STUDY SPECIFIC INFORMED CONSENT FORM
Healthcare Providers

TITLE OF STUDY/PROTOCOL: The CHARM Study-Coordinating transitions from hospital for older adults with fractures: An interventional mixed methods study.

PRINCIPAL INVESTIGATOR: Ian Watson, BSc, MHSc
Administrative Director, Trauma NB
Horizon Health Network, Saint John, NB

FILE NUMBER:

PURPOSE

This study is testing the effects of having support from patient navigators in helping older adults admitted to the Orthopedic Unit with a fracture. The patient navigator will work with the patient's healthcare team to develop a patient specific and attainable care plan needed for a successful discharge from acute care. The study will look to see if there are differences between patients who are assigned patient navigators versus patients receiving usual care (no patient navigator assigned) when it comes to healthcare usage, experiences and satisfaction. The goal of this study is to enhance inpatient care and ensure successful transitions in care for patients and families.

STUDY DESIGN

This study will start June 20, 2022 and finish March 31, 2023. You are being asked to be a part of this study because you are a healthcare provider who was involved in the care of patients assigned to a patient navigator on the Orthopedic Unit at the Saint John Regional Hospital or another receiving facility. We hope to interview 10 health care providers. Patients and family caregivers in both the intervention and standard of care groups will also be interviewed about their experiences in transitioning from acute care.

Someone from the research team will interview you at a time that works for you over the telephone or in person. The interview will take about 20 to 30 minutes. The questions will ask about your experiences with the patient navigators and patients they cared for, what you thought about the role of the patient navigator, and any improvements that could be made to the role of patient navigator. The interview will be audio recorded, then typed up confidentially. All identifying information will be removed.

To be filled out by Person Conducting Verbal Informed Consent Discussion for Research Study Records:

- ☐ Yes, the participant would like to be contacted with the study results.

Contact information: ____

If the participant does not wish to know the results, please check the box below:

- ☐ No, please do not contact the participant.

VOLUNTARY PARTICIPATION

Being interviewed for this study is completely up to you. You can stop at any time, for any reason. There will be no negative repercussions if you decide not to be interviewed. If you decide to stop the interview, you will be asked why but it is up to you whether you provide your reason(s). You may also tell the research team what information that you want them to use and not use in the study.

PRIVACY AND CONFIDENTIALITY

You will be asked about your experiences working alongside a patient navigator and your thoughts on the role. All your information will be kept private and safe. Information will be stored for 7 years once the research is done and the results made public. Information that would identify you will be removed. Only the research team members will be able to read all the information collected. The research team will follow all privacy and confidentiality laws and policies.

The results of the study may be made public. You will not be identified. Your private information will not be shared with others without your permission. Your name will not appear in any writing made public.

RISKS AND POTENTIAL BENEFITS

There are no known risks that you will be exposed to during your voluntary participation in this research study. Potential benefits of this study include helping to understand the impact of support from a patient navigator on inpatient care and patient transitions. This information will be used to inform the development of practical recommendations for policy makers and clinicians on how to enhance inpatient care and successful transitions for older adults.

RESEARCH RELATED INJURY

If you become sick or hurt by taking part in this study, you will receive medical care at no cost to you. By signing this form, you are not waiving your legal rights or freeing the Principal Investigator from their legal responsibilities.

CONTACT INFORMATION

If you have any questions about your rights as someone who takes part in a research study, you may contact the Horizon Health Network Regional Director of Ethics Services at (506)648-6094 or by e-mail at REBOffice@HorizonNB.ca.

If you have any questions or concerns about your privacy rights, you may contact the Privacy Officer for Horizon Health Network at the toll-free number 1-877-422-8717.

Research Study Contact Name:

If you have any questions or concerns about what you just read, please contact the Research Coordinator below:

Leanne Skerry
Research Services, Horizon Health Network
Hilyard Place
560 Main St
Building A, 2nd floor
Suite A-200
E2K 1J5
Phone: 506-643-1321
Email: SOAR@HorizonNB.ca

CONSENT TO PARTICIPATE IN RESEARCH STUDY for *The CHARM Study - Coordinating transitions from hospital for older adults with fractures: An interventional mixed methods study.*

PARTICIPANT'S QUESTIONS:

Have you had an opportunity to ask questions and discuss this study?	☐ Yes	☐ No
Are you comfortable with the information that has been provided?	☐ Yes	☐ No
Do you understand that you are free to withdraw from this study?	☐ Yes	☐ No

PARTICIPANT'S STATEMENT

I have read the above information and understand the purpose of the research. I have had the opportunity to ask questions, and all my questions have been answered. By verbally consenting, I am indicating that I have reviewed all 4 pages of this document. I hereby give my informed consent to be a participant in this study.

To be filled out by Person Conducting Verbal Informed Consent Discussion for Research Study Records:

______________________	______________________	__________
Printed Name of Participant	Verbal Consent of Participant	Date
______________________	______________________	__________
Printed Name of Person Conducting Informed Consent Discussion	Signature of Person Conducting Informed Consent Discussion	Date

Healthcare Provider-Written Consent Form

STUDY SPECIFIC INFORMED CONSENT FORM
Healthcare Providers

TITLE OF STUDY/PROTOCOL: The CHARM Study-Coordinating transitions from hospital for older adults with fractures: An interventional mixed methods study.

PRINCIPAL INVESTIGATOR: Ian Watson, BSc, MHSc
Administrative Director, Trauma NB
Horizon Health Network, Saint John, NB

FILE NUMBER:

PURPOSE

This study is testing the effects of having support from patient navigators in helping older adults admitted to the Orthopedic Unit with a fracture. The patient navigator will work with the patient's healthcare team to develop a patient specific and attainable care plan needed for a successful discharge from acute care. The study will look to see if there are differences between patients who are assigned patient navigators versus patients receiving usual care (no patient navigator assigned) when it comes to healthcare usage, experiences and satisfaction. The goal of this study is to enhance inpatient care and ensure successful transitions in care for patients and families.

STUDY DESIGN

This study will start June 20, 2022 and finish March 31, 2023. You are being asked to be a part of this study because you are a healthcare provider who was involved in the care of patients assigned to a patient navigator on the Orthopedic Unit at the Saint John Regional Hospital or another receiving facility. We hope to interview 10 health care providers. Patients and family caregivers in both the intervention and standard of care groups will also be interviewed about their experiences in transitioning from acute care.

Someone from the research team will interview you at a time that works for you over the telephone or in person. The interview will take about 20 to 30 minutes. The questions will ask about your experiences with the patient navigators and patients they cared for, what you thought about the role of the patient navigator, and any improvements that could be made to the role of patient navigator. The interview will be audio recorded, then typed up confidentially. All identifying information will be removed.

If you want, the results of the study will be shared with you. This can help make sure that the findings are correct. If you would like to find out the results of the study, please check the box below:

- □ Yes, I would like to be contacted with the study results.

Contact information: ____

If you do not wish to know the results, please check the box below:

- □ No, please do not contact me.

VOLUNTARY PARTICIPATION

Being interviewed for this study is completely up to you. You can stop at any time, for any reason. There will be no negative repercussions if you decide not to be interviewed. If you decide to stop the interview, you will be asked why but it is up to you whether you provide your reason(s). You may also tell the research team what information that you want them to use and not use in the study.

PRIVACY AND CONFIDENTIALITY

You will be asked about your experiences working alongside a patient navigator and your thoughts on the role. All your information will be kept private and safe. Information will be stored for 7 years once the research is done and the results made public. Information that would identify you will be removed. Only the research team members will be able to read all the information collected. The research team will follow all privacy and confidentiality laws and policies.

The results of the study may be made public. You will not be identified. Your private information will not be shared with others without your permission. Your name will not appear in any writing made public.

RISKS AND POTENTIAL BENEFITS

There are no known risks that you will be exposed to during your voluntary participation in this research study. Potential benefits of this study include helping to understand the impact of support from a patient navigator on inpatient care and patient transitions. This information will be used to inform the development of practical recommendations for policy makers and clinicians on how to enhance inpatient care and successful transitions for older adults.

RESEARCH RELATED INJURY

If you become sick or hurt by taking part in this study, you will receive medical care at no cost to you. By signing this form, you are not waiving your legal rights or freeing the Principal Investigator from their legal responsibilities.

CONTACT INFORMATION

If you have any questions about your rights as someone who takes part in a research study, you may contact the Horizon Health Network Regional Director of Ethics Services at (506)648-6094 or by e-mail at REBOffice@HorizonNB.ca.

If you have any questions or concerns about your privacy rights, you may contact the Privacy Officer for Horizon Health Network at the toll-free number 1-877-422-8717.

Research Study Contact Name:

If you have any questions or concerns about what you just read, please contact the Research Coordinator below:

Leanne Skerry
Research Services, Horizon Health Network
Hilyard Place
560 Main St
Building A, 2nd floor
Suite A-200
E2K 1J5
Phone: 506-643-1321
Email: SOAR@HorizonNB.ca

CONSENT TO PARTICIPATE IN RESEARCH STUDY for *The CHARM Study - Coordinating transitions from hospital for older adults with fractures: An interventional mixed methods study.*

PARTICIPANT'S QUESTIONS:

Have you had an opportunity to ask questions and discuss this study?	☐ Yes	☐ No
Are you comfortable with the information that has been provided?	☐ Yes	☐ No
Do you understand that you are free to withdraw from this study?	☐ Yes	☐ No
Do you understand that you will receive a signed copy of this consent?	☐ Yes	☐ No

PARTICIPANT'S STATEMENT
I have read the above information and understand the purpose of the research. I have had the opportunity to ask questions, and all my questions have been answered. By signing, I am indicating that I have reviewed all 4 pages of this document. I hereby give my informed consent to be a participant in this study.

Printed Name of Participant	Signature of Participant	Date

Printed Name of Person Conducting Informed Consent Discussion	Signature of Person Conducting Informed Consent Discussion	Date

Appendix C

Participant Assent Letter

The CHARM Study-Coordinating transitions from hospital for older adults with fractures: An interventional mixed methods study

Assent Summary

This is a study to find out about how having support from patient navigators helps older adults admitted to the Orthopedic Unit with a fracture. We will ask you questions about being in the hospital and then how you are doing after you are discharged from the Orthopedic unit.

Statement of Assent

I have decided to be in the study even though I know that I don't have to do it. The researcher has answered all my questions.

______________________________________ ________________
Printed Name of Participant Age

______________________________________ ________________
Signature of Participant (if capable of signing) Date

STATEMENT OF PERSON EXPLAINING ASSENT
To be completed by the person explaining assent (please check one):

- □ Participant read assent summary independently.
- □ Participant was read the information contained in the summary form.

I have carefully explained to the participant and the participant's substitute decision maker the nature and purpose of the above study. There has been an opportunity for the participant and the participant's substitute decision maker to ask questions about this research study. I have been available to answer any questions that the participant and the participant's substitute decision maker has about this study.

____ ____________________
Signature of Person Explaining Assent Date

____
Printed Name of Person Explaining Assent

Appendix D

Participant Semi-Structured Interview Guides

Patient

1. Why were you admitted to the hospital three months ago?
2. How did you feel about being in the hospital?
3. Can you tell me about the care you had in the hospital?
4. Did you feel like you were actively involved in decisions about your care? Can you tell me why or why not?
5. Can you tell me about your discharge from the orthopedic unit? Where were you discharged to?
6. What sort of information were you given about your discharge from the orthopedic unit?
    a. Who told you this information?
    b. What sort of supports or help did you have set up?
    c. Did you have any help before you were admitted to the hospital?
    d. How were you involved in the discharge plans?
7. How did you feel about being discharged from the orthopedic unit?
    a. Did you have any concerns?
    b. Did you feel prepared?
8. How have you been managing since discharge from the orthopedic unit? [Probes used as appropriate depending on patient circumstances.]
    a. How are you making out with the medications?
    b. How are you doing with the other instructions on your discharge plan?
    c. What kind of support is there for home care?
        i. Has this changed?
    d. How are you managing daily activities?
        i. Can you tell me about an average day?
    e. How have you been feeling?
        i. Is there anything worrying you?
        ii. What has been going well?
9. Can you tell me about your experiences with the Patient Navigator [name of assigned patient navigator]? ***(Intervention Group Only)***

   a. How were they helpful?
   b. What could have been improved about the care you received?

10. Is there anything you would have liked to happen differently in being discharged from the orthopedic unit?
    a. What could have made the transition easier?
    b. What was helpful?
11. Is there anything else we should know about your experiences in the hospital and being discharged from the orthopedic unit?
    a. ***(Intervention Group Only)*** Is there anything else we should know about your experiences with the patient navigator?

Family Caregiver

1. What is your relationship with [patient's name]?
2. What kind of help did you give them before they were admitted to the hospital?
   a. How many hours a week did you help them?
   b. Did [patient's name] have anyone else helping them?
3. Can you tell me about [patient's name] discharge from the orthopedic unit? Where were they discharged to? [Prompts used depending on patient circumstances.]
   a. What sort of information were you given?
   b. Who told you this information?
   c. What sort of supports or help were set up for them?
   d. How have you been helping [patient's name] since they've been discharged from the orthopedic unit?
4. How did you feel about [patient's name] being discharged from the orthopedic unit?
   a. Did you have any concerns?
   b. Did you feel prepared?
5. Do you have any concerns about your own health?
6. How have you been managing since discharge from the orthopedic unit? [Probes used as appropriate depending on patient circumstances.]

a. How have you been making out caring for them since leaving the hospital?

b. Do you have anyone supporting you?

c. Are there other supports for [patient's name] in place?

   i. What kind of support is there for home care?
      1. Has this changed?

d. How has your health been?

e. How have you been feeling?
   i. Is there anything worrying you?
   ii. What has been going well?
f. How is [patient's name] making out with their medications?
g. How is [patient name] doing with the other instructions on their discharge plan?
h. How is [patient name] managing daily activities?
   i. Can you tell me about an average day?

7. Can you tell me about your and [patient's name] experiences with the Patient Navigator [name of assigned patient navigator]? ***(Intervention Group Only)***

   a. How were they helpful?

   b. What could have been improved about the care you received?

8. Is there anything you would have liked to happen differently in being discharged from the orthopedic unit?

   a. What could have made the transition easier?

   b. What was helpful?

9. Is there anything else we should know about [patient's name] experiences in the hospital and being discharged from the orthopedic unit?

   a. ***(Intervention Group Only)*** Is there anything else we should know about your experiences with the patient navigator?

Healthcare Provider

1. Can you tell me what kind of healthcare provider you are and how you were involved with patient care for those assigned a patient navigator?

2. What were your experiences with the patient navigators?

   a. How did they help with patient transitions and discharge?

b. What issues arose working with patient navigators, if any?

3. What improvements could be made to the role of the patient navigators?

4. What do you think of the role of patient navigators?

5. Is there anything else you would like to say regarding patient navigators?

Appendix E

Study Participation Notification Letter

Date
Name
Organization
Address, Address Address

SUBJECT: Patient Research Participation

Dear Dr. [Name],

This letter is to inform you of [Patient's Name]'s participation in the research project, *The CHARM Study- Coordinating transitions from hospital for older adults with fractures: An interventional mixed methods study*. They have given informed consent to be a participant in the research project. [or- Informed consent has been given for their participation by [Name of Substitute Decision Maker], as the Substitute Decision Maker]. This project has been institutionally approved by the Horizon Health Network Human Research Protection Program (HRPP), which is inclusive of the Research Ethics Board, [REB File #:].

The *CHARM* project is investigating the effects of having support from patient navigators in helping older adults admitted to the Orthopedic Unit with a fracture. The patient navigator will work alongside the patient's healthcare team to develop a patient specific and attainable care plan needed for a successful discharge from acute care. The goal of this study is to enhance inpatient care and ensure successful transitions in care for patients and families.

Participation in this project entails that [Patient's Name] will be followed through their transition from the Orthopedic unit (OU), with participation ending three months after discharge from the OU. This is a mixed-methods study about the transition process, and data will be collected on [Patient's Name] via surveys, medical records and a semi-structured interview. Health care information will be collected regarding discharge care plans and follow-up appointments, as well as self-reported health care utilization from discharge to three months.

If you have any questions or concerns about the project please feel free to contact myself as Principal Investigator, the Horizon Health Network Research Ethics Board with ethical concerns (REBOffice@Horizonnb.ca or 506-648-6094), or the University of New Brunswick Research Ethics Board (506-648-5908).

Regards,

Ian Watson, BSc, MHSc
Administrative Director, Trauma NB
Horizon Health Network
400 University Avenue, Saint John, NB E2L 4L2
506-648-8042
Ian.Watson@HorizonNB.ca

References

1. Canada S. Demographic estimates by age and sex, provinces and territories. 2021.

2. Naylor MD, Aiken LH, Kurtzman ET, Olds DM, Hirschman KB. The importance of transitional care in achieving health reform. *Health affairs.* 2011;30(4):746-754.
3. Weeks L, Barber B, MacDougall ES, et al. Supporting the Transition From Hospital to Home for Older Adults: Case Study Results. *Innovation in Aging.* 2020;4(Supplement_1):81-81.
4. Naylor MD. Transitional care of older adults. *Annual review of nursing research.* 2002;20(1):127-147.
5. Glasby J. *Understanding health and social care.* Policy Press; 2017.
6. Subbe C, Goulden N, Mawdsley K, Smith R. Anticipating care needs of patients after discharge from hospital: Frail and elderly patients without physiological abnormality on day of admission are more likely to require social services input. *European journal of internal medicine.* 2017;45:74-77.
7. Salehi V, Hanson N, Smith D, McCloskey R, Jarrett P, Veitch B. Modeling and analyzing hospital to home transition processes of frail older adults using the functional resonance analysis method (FRAM). *Applied Ergonomics.* 2021;93:103392.
8. Skerry L, Kervin, E., Hanson, Jarrett, P., McCloskey, R. Using a social ecological approach to understand transitions from hospital to home for frail older adults. *Canadian Geriatrics Society 40th Annual Scientific Meeting.* 2021.
9. Coleman EA, Parry C, Chalmers S, Min S-j. The care transitions intervention: results of a randomized controlled trial. *Archives of internal medicine.* 2006;166(17):1822-1828.
10. Jack BW, Chetty VK, Anthony D, et al. A reengineered hospital discharge program to decrease rehospitalization: a randomized trial. *Annals of internal medicine.* 2009;150(3):178-187.
11. Freeman HP, Rodriguez RL. History and principles of patient navigation. *Cancer.* 2011;117(S15):3537-3540.
12. Freeman HP. The origin, evolution, and principles of patient navigation. In. Vol 21: AACR; 2012:1614-1617.
13. Kohr R. Patient Navigation in a patient-centered, collaborative world. Paper presented at: Canadian Healthcare Navigation Conference2021.
14. Kelly K, Doucet S, Luke A. Exploring the roles, functions, and background of patient navigators and case managers: A scoping review. *International journal of nursing studies.* 2019;98:27-47.
15. Paskett ED, Harrop JP, Wells KJ. Patient navigation: an update on the state of the science. *CA: a cancer journal for clinicians.* 2011;61(4):237-249.
16. Samm M, Gibson A. Developing a conceptual patient navigation framework: A standardized approach to support patients and families through their healthcare journey. In: Markham Stouffville Hospital; n.d.
17. Clarfield AM, Bergman H, Kane R. Fragmentation of care for frail older people—an international problem. Experience from three countries: Israel, Canada, and the United States. *Journal of the American Geriatrics Society.* 2001;49(12):1714-1721.
18. Manderson B, Mcmurray J, Piraino E, Stolee P. Navigation roles support chronically ill older adults through healthcare transitions: a systematic review of the literature. *Health & social care in the community.* 2012;20(2):113-127.
19. Hajewski CJ, Shirey MR. Care coordination. *The Journal of Nursing Administration.* 2014;44(11):577-585.
20. Verhaegh KJ, MacNeil-Vroomen JL, Eslami S, Geerlings SE, de Rooij SE, Buurman BM. Transitional care interventions prevent hospital readmissions for adults with chronic illnesses. *Health affairs.* 2014;33(9):1531-1539.

21. Balaban RB, Galbraith AA, Burns ME, Vialle-Valentin CE, Larochelle MR, Ross-Degnan D. A patient navigator intervention to reduce hospital readmissions among high-risk safety-net patients: a randomized controlled trial. *Journal of General Internal Medicine.* 2015;30(7):907-915.
22. Horne HN, Phelan-Emrick DF, Pollack CE, et al. Effect of patient navigation on colorectal cancer screening in a community-based randomized controlled trial of urban African American adults. *Cancer Causes Control.* 2015;26(2):239-246.
23. Campbell C, Craig J, Eggert J, Bailey-Dorton C. Implementing and measuring the impact of patient navigation at a comprehensive community cancer center. Paper presented at: Oncology Nursing Forum2010.
24. Luke A, Doucet S, Azar R. Paediatric patient navigation models of care in Canada: an environmental scan. *Paediatrics & Child Health.* 2018;23(3):e46-e55.
25. Public Health Agency of Canada. Seniors' falls in Canada: Second report. In:2014.
26. Trauma NB. NB Trauma Registry. In:2021.
27. Clegg A, Young J, Iliffe S, Rikkert MO, Rockwood K. Frailty in elderly people. *The Lancet.* 2013;381(9868):752-762.
28. CFN. Frailty in Canada: What is Frailty. Canadian Frailty Network. http://www.cfn-nce.ca/frailty-in-canada/. Accessed.
29. Canadian Frailty Network. What is Frailty? https://www.cfn-nce.ca/frailty-matters/what-is-frailty/#:~:text=Frailty%20is%20a%20medical%20condition,multiple%20medical%20conditions%20and%20frailty. Published 2021. Accessed May 10th 2021.
30. Chen C-L, Chen C-M, Wang C-Y, et al. Frailty is associated with an increased risk of major adverse outcomes in elderly patients following surgical treatment of hip fracture. *Scientific reports.* 2019;9(1):1-9.
31. Ensrud KE, Ewing SK, Taylor BC, et al. Frailty and risk of falls, fracture, and mortality in older women: the study of osteoporotic fractures. *The Journals of Gerontology Series A: Biological Sciences and Medical Sciences.* 2007;62(7):744-751.
32. Hoover M, Rotermann M, Sanmartin C, Bernier J. Validation of an index to estimate the prevalence of frailty among community-dwelling seniors. *Health Rep.* 2013;24(9):10-17.
33. Mehrabi F, Béland F. Effects of social isolation, loneliness and frailty on health outcomes and their possible mediators and moderators in community-dwelling older adults: A scoping review. *Archives of Gerontology and Geriatrics.* 2020;90:104119.
34. Kojima G. Frailty defined by FRAIL scale as a predictor of mortality: a systematic review and meta-analysis. *Journal of the American Medical Directors Association.* 2018;19(6):480-483.
35. Horizon Health Network. Horizon’s Strategic Plan 2021-2026: Building a Healthier Future. In: Network HH, ed: Horizon Health Network; 2021.
36. Government of New Brunswick. Striving for Dependable Public Health Care: A discussion paper on the future of health care in New Brunswick. In:2021.
37. Theou O, Andrew M, Ahip SS, et al. The pictorial fit-frail scale: developing a visual scale to assess frailty. *Canadian Geriatrics Journal.* 2019;22(2):64.
38. Wallace LM, McGarrigle L, Rockwood K, Andrew MK, Theou O. Validation of the pictorial fit-frail scale in a memory clinic setting. *International Psychogeriatrics.* 2020;32(9):1063-1072.
39. McGarrigle L, Squires E, Wallace LM, et al. Investigating the feasibility and reliability of the Pictorial Fit-Frail Scale. *Age and Ageing.* 2019;48(6):832-837.

40. Ysea-Hill O, Sani TN, Nasr LA, et al. Concurrent Validity of Pictorial Fit-Frail Scale (PFFS) in Older Adult Male Veterans with Different Levels of Health Literacy. *Gerontology and Geriatric Medicine.* 2021;7:23337214211003804.
41. Owens B. Survey aims to capture patient experience. In: Can Med Assoc; 2015.
42. Hadibhai S, Lacroix J, Leeb K. Developing the first pan-Canadian acute care patient experiences survey. *Patient Experience Journal.* 2018;5(3):25-33.
43. Nadin S, Miandad MA, Kelley ML, Marcella J, Heyland DK. Measuring family members' satisfaction with end-of-life care in long-term care: Adaptation of the canhelp lite questionnaire. *BioMed Research International.* 2017;2017.
44. Heyland DK, Jiang X, Day AG, Cohen SR, Network CRatEoL. The development and validation of a shorter version of the Canadian Health Care Evaluation Project Questionnaire (CANHELP Lite): a novel tool to measure patient and family satisfaction with end-of-life care. *Journal of pain and symptom management.* 2013;46(2):289-297.
45. Creswell JW. *Research Design: Qualitative, Quantitative, and Mixed Methods Approaches.* SAGE Publications; 2009.
46. Tabachnick BG, Fidell LS. *Using Multivariate Statistics.* New Jersey: Pearson Education; 2013.
47. Breitsohl H. Beyond ANOVA: An introduction to structural equation models for experimental designs. *Organizational Research Methods.* 2019;22(3):649-677.
48. Van de Schoot R, Miocević M. *Small sample size solutions: A guide for applied researchers and practitioners.* Taylor & Francis; 2020.
49. Rosseel Y. Small sample solutions for structural equation modeling. In: *Small sample size solutions: A guide for applied researchers and practitioners.* Routledge; 2020:226-238.
50. Braun V, Clarke V. Using thematic analysis in psychology. *Qualitative Research in Psychology.* 2006;3(2):77-101.
